# Virulence Pattern and Genomic Diversity of Vibrio cholerae O1 and O139 Strains Isolated From Clinical and Environmental Sources in India

**DOI:** 10.3389/fmicb.2020.01838

**Published:** 2020-08-26

**Authors:** Amol Kanampalliwar, Durg Vijai Singh

**Affiliations:** ^1^Department of Infectious Disease Biology, Institute of Life Sciences, Bhubaneswar, India; ^2^Department of Biotechnology, School of Earth, Biological and Environmental Sciences, Central University of South Bihar, Gaya, India

**Keywords:** *Vibrio cholerae*, virulence, MLST, PFGE, sequence type, diversity

## Abstract

*Vibrio cholerae* is an autochthonous inhabitant of the aquatic environment. Several molecular methods have been used for typing *V. cholerae* strains, but there is no proper database for such scheme, including multilocus sequence typing (MLST) for *V. cholerae* O1 and O139 strains. We used 54 *V. cholerae* O1 and three O139 strains isolated from clinical and environmental sources and regions of India during the time period of 1975–2015 to determine the presence of virulence genes and production of biofilm. We devised a MLST scheme and developed a database for typing *V. cholerae* strains. Also, we performed pulsed-field gel electrophoresis to see the genomic diversity among them and compared it with MLST. We used the MEGA 7.0 software for the alignment and comparison of different nucleotide sequences. The advanced cluster analysis was performed to define complexes. All strains of *V. cholerae*, except five strains, showed variation in phenotypic characteristics but carried virulence-associated genes indicating they belonged to the El Tor/hybrid/O139 variants. MLST analysis showed 455 sequences types among *V. cholerae* strains, irrespective of sources and places of isolation. With these findings, we set up an MLST database on PubMLST.org using the BIGSdb software for *V. cholerae* O1 and O139 strains, which is available at https://pubmlst.org/vcholerae/ under the O1/O139 scheme. The pulsed-field gel electrophoresis (PFGE) fingerprint showed six fingerprint patterns namely E, F, G, H, I, and J clusters among 33 strains including strain N16961 carrying El Tor *ctxB* of which cluster J representing O139 strain was entirely different from other El Tor strains. Twenty strains carrying Haitian *ctxB* showed a fingerprint pattern classified as cluster A. Of the five strains, four carrying classical *ctxB* comprising two each of El Tor and O139 strains and one El Tor strain carrying Haitian *ctxB* clustered together under cluster B along with *V. cholerae* 569B showing pattern D. This study thus indicates that *V. cholerae* strains are undergoing continuous genetic changes leading to the emergence of new strains. The MLST scheme was found more appropriate compared to PFGE that can be used to determine the genomic diversity and population structure of *V. cholerae.*

## Introduction

*Vibrio cholerae* is an autochthonous inhabitant of the aquatic environment such as brackish water and estuaries and exists in association with zoo- and phyto-planktons (Colwell et al., 1977). Of the over 206 serogroups, only O1 and O139 *V. cholerae* are responsible for causing epidemics and pandemics (Lee et al., 2006); the non-O1 and non-O139 serogroups are associated with sporadic outbreaks of cholera and in extra-intestinal infections (Hughes et al., 1978; Blake et al., 1980; Gelbart and Prabhudesai, 1986; Safrin et al., 1988). The O1 serogroup is classified into two biotypes, Classical and El Tor, depending on phenotypic and genotypic differences and three serotypes Ogawa, Inaba, and Hikojima (Kaper et al., 1995).

*Vibrio cholerae* contains two chromosomes and possesses islands that are responsible for the adherence, synthesis, and production of the cholera toxin (Kirn et al., 2000). Also, this organism possess other virulence factors like accessory cholera enterotoxin (*ace*), zonula occludens toxin (*zot*), repeat toxin (*rtxA*), hemolysin (*hlyA*), mannose-sensitive hemagglutinin (*mshA)*, and the lipopolysaccharide biosynthesis gene (*rfb*) of O1/O139 responsible for the pathogenesis of cholera (Waldor and Mekalanos, 1996; Menezes et al., 2014). However, the type III secretion system (T3SS) plays a vital role in the pathogenesis of non-O1 and non-O139 *V. cholerae* (Luo et al., 2013).

There are reports of the replacement of prototypic El Tor strains with El Tor variants in several countries which cause more severe cholera with a high rate of fatality (Nair et al., 2002; Raychoudhuri et al., 2008; Chin et al., 2011; Talkington et al., 2011; Hasan et al., 2012; Das et al., 2016). In this context, several molecular biology techniques have been used to study the genomic diversity of *V. cholerae* strains (Kotetishvili et al., 2003). Multilocus sequence typing (MLST), a discriminant DNA-based typing method, has been utilized to analyze the bacterial strains (Garg et al., 2003). Although MLST with 3–9 housekeeping genes was used to type *V. cholerae* strains (Garg et al., 2003; Kotetishvili et al., 2003; Lee et al., 2006; Mironova et al., 2015; Liao et al., 2018), there is no proper MLST database for typing *V. cholerae* O1 and O139 strains, except for non-O1, non-O139 strains (Octavia et al., 2013). In the present study, we characterized *V. cholerae* O1 and O139 strains isolated during the time period 1975–2015 from clinical and environmental sources of India for the presence of virulence genes and tested them for their ability to produce biofilm. We also devised an MLST scheme for typing *V. cholerae* O1 and O139 strains and developed a database as https://pubmlst.org/v.cholerae to see the genomic diversity and compared them with pulsed-field gel electrophoresis (PFGE).

## Materials and Methods

### Bacterial Strains

Of the total 54 *V. cholerae* O1 strains, of which 50 clinical, four environmental, and three clinical O139 strains from laboratory stock were included in the study. These strains were from different regions of India and isolated during 1975–2015. The strains were previously identified using standard bacteriological and serological methods (World Health Organization [WHO], 1987). Also, *V. cholerae* strains N16961, 569B, and MO10 were used as controls in the study. All strains were tested for the oxidase reaction, and the identities of the *V. cholerae* O1 strains were confirmed by serogrouping with polyvalent O1 and mono-specific Inaba and Ogawa antisera (Denka Seiken, Co., Ltd., Japan). *V. cholerae* strains that did not agglutinate with O1 antiserum were checked with monoclonal O139 antiserum supplied by the WHO Regional Office for South-East Asia, New Delhi, India. Biotyping of *V. cholerae* O1 strains was performed by the method described earlier (Das et al., 2016). All strains were maintained in Tryptic soy broth with 20% glycerol at −80°C. The details of the strains are given in Table 1.

**TABLE 1:** Phenotypic and genotypic characterization of *Vibrio cholerae* O1 and O139 strains used in this study from clinical and environmental sources in India.

***V. cholerae* strains**	**Phenotype(s)**				**Genotype(s)**		**Biotype(s)**	**Serotype(s)**
	**CCA**	**Hly**	**VP**	**PB (50U)**	***ctxB***	***rstR***		
TVM227, TVM 182	+	−	+	R	C	C	O139 variant I	O139
TVM 261	+	−	+	S	ET	E	O139 variant II	
V1, V20, 2179, TVM149	−	+	+	R	ET	E	El Tor variant III	O1
RHV16	−	+	+	S	ET	E	El Tor variant IV	
AL40, 10, KO194, VKO12, MO100	+	+	+	R	ET	E	El Tor	
32T, KO211	+	+	+	S	ET	E	El Tor variant V	
TV123, TV281, IDH5/89, CH486, 10T	−	+	+	R	ET	E	El Tor variant VI	
814B	−	+	+	S	ET	E	El Tor variant VII	
TVM152, TVM131, TVM134	−	−	+	R	ET	E	El Tor variant VIII	
TVM137	−	−	+	S	ET	E	El Tor variant IX	
RHV450, NICED1, NICED9, 381, 224	+	+	−		ET	E	El Tor variant X	
TV10, TV110, DO183	+	−	+	R	ET	E	El Tor variant XI	
MO108	+	−	+	S	ET	E	El Tor variant XII	
DN3	+	+	+	R	C	E	El Tor variant I	
DN56	−	−	−	S	H	E	El Tor variant II	
DN47	+	+	+	R	H	E	Hybrid variant I	
DN64	−	+	+	R	H	E	Hybrid variant II	
DN4, DN36, DN55	+	+	−	S	H	E	Hybrid variant III	
DN5, DN65	+	+	+	S	H	E	Hybrid variant IV	
DN19	−	+	+	S	H	E	Hybrid variant IV	
DN13, DN52, DN73	+	−	+	R	H	E	Hybrid variant V	
DN17	−	+	−	R	C	E	Hybrid variant VI	
DN34, DN54	+	−	+	S	H	E	Hybrid variant VII	
DN23	−	−	−	R	H	E	Hybrid variant VIII	
DN69	+	+	−	R	H	E	Hybrid variant X	
DN51, DN74	+	−	−	R	H	E	Hybrid variant XI	
DN50	+	−	−	S	H	E	Hybrid variant XII	
DN78, DN81	−	−	+	S	H	E	Hybrid variant XIII	O1

*CCA, chick erythrocyte cell agglutination; Hly, sheep erythrocyte hemolysis; VP, voges-proskauer; PB, Polymyxin B (50U); *ctxB*, cholera toxin subunit B; *rstR*, repeat sequence transcriptional regulator.*

### PCR Assays

*Vibrio cholerae* strains grown overnight at 37°C in Luria-Bertani (LB) broth (Difco) were boiled for 10 min and stored at -20°C until use. The bacterial cell lysates were used as template DNA in all polymerase chain reaction (PCR) assays. Hexaplex PCR determined the presence of virulence and regulatory genes *ctxA*, *zot*, *ace*, *tcpA*, *ompU*, and *toxR* (Singh et al., 2002). Single PCR assays were performed to detect the *tcpI*, *ctxB*, *rstC, rtxA*, and *hlyA* genes (Singh et al., 2001). Other PCR assays were also used to examine the presence of *rstR*, Vibrio seventh pandemic island I (VSP-I) and the seventh pandemic group-specific marker V2346, and to examine for insertion of CTXΦ and VSP-I in the chromosomes (Maiti et al., 2006; Grim et al., 2010). Amplification was performed using oligonucleotides (GCC Biotech Pvt., Ltd., New Delhi, India) as shown in Supplementary Table S1. Amplified products were separated on agarose gel, stained with ethidium bromide, and visualized in Fluoro-S MultiImager (Bio-Rad Laboratories, Inc., United States).

### DMAMA- and MAMA-PCR

We used the double mismatch amplification mutation assay (DMAMA)- PCR for detection of classical, and Haitian *ctxB* using biotype specific primers Rv-cla (5’-CCT GGT ACT TCT ACT TGA AAC G-3’); ctxB-3 (5’-GTT TTA CTA TCT TCA GCA TAT GCG A-3’); ctxB-4 (5’-GTT TTA CTA TCT TCA GCA TAT GCG C-3’ (Naha et al., 2012). MAMA-PCR was also performed using primers Fw-com (5’-ACT ATC TTC AGC ATA TGC ACA TGG-3’) and Re-elt (5’-CCT GGT ACT TCT ACT TGA AAC A-3’) to see the presence of El Tor *ctxB* in these strains (Morita et al., 2008). Amplification was performed using oligonucleotides (GCC Biotech Pvt., Ltd., New Delhi, India) and PCR condition as shown in Supplementary Table S1.

### Assay for Biofilm Formation

Biofilm in *V. cholerae* O1 and O139 strains was quantified by the method given by O’Toole (2011). Briefly, overnight grown cultures of *V. cholerae* O1 strains normalized to 1-OD at 630 nm were diluted 100 times and inoculated in fresh LB contained in 96-well flat bottom polystyrene plates. After 24 h of incubation at 30°C under static condition, the culture was removed, and the plate was washed with water. Adherent cells were then stained with 0.1% crystal violet, washed thoroughly with water and dissolved in dimethyl sulfoxide. The formation of biofilm was measured at OD 540 nm in ELISA Reader (Variscan, Thermo Fisher Scientific) using the *V. cholerae* O1 strain N16961 and *Escherichia coli* strain DH5α as positive and negative controls, respectively. Biofilm formation experiments were done in triplicate, and the data showing values of OD 540 nm > 0.05 were omitted. The temporal effect of DNase I (0.5 mg/ml; Sigma, DN25), NaIO4 (40 mM; Sigma, S1878), and proteinase K (0.1 mg/ml; Sigma, P2308) was conducted to determine the components of the biofilm matrix (Hall-Stoodley et al., 2008).

### Multilocus Sequence Typing (MLST) and Phylogenetic Analysis

The MLST of *V. cholerae* strains was performed using PCR with primers (Supplementary Table S2) and obtained with five housekeeping genes, namely, aspartate-semialdehyde dehydrogenase (*asd*), DNA polymerase III α-holoenzyme (*dnaE*), leucine aminopeptidase (*lap*), phosphoglucomutase (*pgm*), and recombinase repair protein A (*recA*) with some modification (Garg et al., 2003). Amplification was performed using oligonucleotides (GCC Biotech Pvt., Ltd., New Delhi, India) as shown in Supplementary Table S2. The PCR products were purified using ExoSAP (Affymetrix, Inc., Cleveland, OH, United States). Both strands were sequenced using the ABI sequencer model 3500 (Life Technologies Holdings Pte Ltd., Marsiling, Singapore) at the sequencing facility of the Institute of Life Sciences (Bhubaneswar, India). The nucleotide sequences were then aligned with MEGA 7.0 software and new alleles designated. Sequences were performed in biological duplicate to confirm the presence of new alleles. A unique allele number was assigned to each sequence even if they differed at a single nucleotide site sequentially, and no weighting was applied to reflect the number of nucleotide differences between alleles.

The advanced cluster analysis was performed to define the clonal complexes (CC) by using the BioNumerics software, version 7.6 (Applied Maths, Belgium). A minimum spanning tree (MST) was constructed using the MLST data, and partitions were created to form clusters. The similarity in at least three alleles grouped isolates of *V. cholerae* in one CC. The central ST of each partition was used to designate a CC. The DnaSP program was used to determine polymorphic, informative, singleton synonymous, and non-synonymous sites (Rozas et al., 2003).

### MLST Database for *V. cholerae* O1 and O139

The MLST database for *V. cholerae* O1 and O139 was created on the PubMLST.org website using the BIGSdb software and made available at https://pubmlst.org/vcholerae/ to assign new STs among *V. cholerae* O1 and O139 strains (Jolley and Maiden, 2010).

### Nucleotide Sequence Accession Numbers

All the sequences were submitted to GenBank with accession numbers as MH169789–MH169845 for *asd*, MH169846–MH169902 for *dnaE*, MH232265–MH232321 for *lap*, MH232322–MH232378 for *pgm*, and MH232379–MH232435 for *recA*, respectively.

### Pulsed-Field Gel Electrophoresis and Image Analysis

*Not*I restriction enzyme (New England Biolabs, United Kingdom) was used to analyze strains using a Pulse Net standardized PFGE protocol for *V. cholerae* (Cooper et al., 2006). The digested fragments were separated in 1% agarose (PFGE grade, Bio-Rad Laboratories, Inc.) prepared in a 0.5X TBE buffer in Clamped Homogenous Electric Fields Mapper (Bio-Rad Laboratories, Inc.). A standard running condition of the PulseNet protocol l^1^ was used for the separation of DNA fragments. The gel was stained with ethidium bromide and photographed in a gel documentation system (Bio-Rad Laboratories, Inc., United States).

After visualization, the fingerprint profile in the PFGE banding pattern was analyzed by using the computer software package BioNumerics (7.1 versions) (Applied Maths, Belgium). The fingerprinting pattern, after background subtraction and gel normalization, was subjected to typing by band-based similarity Dice coefficient, which provides a quantitative assessment of strain similarity. Strains were clustered together using 1.5% optimization, 1.5% tolerance, and a threshold linkage value of >95% similarity matrix. Clustering was done based on unweighted-pair group methods using average linkages (UPGMA) as recommended by the software manufacturer, and results are graphically represented as a dendrogram.

## Results

### Phenotypic Characteristics

Of the 57 *V. cholerae*, 54 strains belonged to serogroup O1, and three strains belonged to serogroup O139. Of the 54 O1 strains, six strains were serotype Inaba and the remaining strains were serotype Ogawa. Five strains isolated from Kerala were positive for Voges–Proskauer (VP) test, hemolysis, and chicken cell erythrocyte agglutination (CCA), and were resistant to polymyxin B. All these strains carried the *ctxB* gene; however, one strain from Silvassa that showed typical characteristics of El Tor and carried the classical *ctxB* gene was designated as El Tor variant-I (Table 2). Twenty-seven strains, although carrying the El Tor *ctxB* gene, showed variable results for VP test, hemolysis, CCA, and polymyxin B sensitivity and were classified as El Tor variants II–XII. Twenty-one strains of *V. cholerae* O1 mostly isolated from Silvassa showed variable results for VP test, hemolysis, CCA, and polymyxin B sensitivity but carried the Haitian *ctxB* gene, Except for one strain that carried classical *ctxB* gene, they were designated as variants I–XII (Table 2). Of the three, two strains of *V. cholerae* O139 that carried classical *ctxB*, and one strain that carried El Tor *ctxB* but showed variable results for VP test, hemolysis, and CCA were designated as O139 variants I–II (Table 2).

**TABLE 2:** Sequence variations of different genetic loci of five housekeeping genes obtained with *V. cholerae* O1 and O139 strains by DnaSP.

**Gene**	**Fragment size (bp)**	**Total Number of Sites**				
		**Polymorphic site**	**Informative site**	**Singleton sites**	**Synonymous sites**	**Non-synonymous sites**
*asd*	543	71	16	33	57	13
*dnaE*	502	83	23	41	65	16
*lap*	479	69	9	19	53	14
*pgm*	553	101	65	23	94	6
*recA*	686	47	36	1	34	10

**asd*, aspartate-semialdehyde dehydrogenase; *dnaE*, DNA polymerase III α-holoenzyme; *lap*, leucine aminopeptidase; *pgm*, phosphoglucomutase; *recA*, recombinase repair protein A.*

### Virulence and Regulatory Genes

All the 57 *V. cholerae* strains harbored *ctxA*, *ompU* and *tcpAI*, *zot, ace*, and *toxR* genes, except three isolates (TV10, 814B, and IDH5/89) that lacked *zot* and *ace* genes, and one strain that lacked the *toxR* gene (data not shown). DMAMA-PCR showed that four strains (TVM227, TVM182, DN3, and DN17) carried the classical prototype of *ctxB* similar to classical *V. cholerae* 569B; 31 strains carried the El Tor prototype of *ctxB*, and 22 strains harbored the Haitian prototype *ctxB*. All these strains also carried *tcpI, rtxC, rtxA, hlyA^*ET*^*, and hly^Class^ genes. Two strains (TVM182, TVM 227) carried the *rstR*^*class*^ and 55 isolates carried *rstR*^*El Tor*^ (Table 1).

### Vibrio Seventh Pandemic Island I

*Vibrio cholerae* strains N16961 harbor VSP-I in between VC0174 and VC0186 on large chromosome. However, strain MJ-1236 contains VSP-I on both chromosomes (Grim et al., 2010). In addition, all strains amplified the fragment of VC0175 showing the presence of VSP-I. All strains of *V. cholerae* O1 and O139 contained VSP-I integrated only on large chromosome and yielded the 1321 bp fragment with chromosome I and VSP-I specific PCR (data not shown). All these strains also carried the seventh pandemic-specific marker VC2346 which amplified a 405 bp fragment.

### Biofilm Formation and Detachment Analysis

**FIGURE 1:**
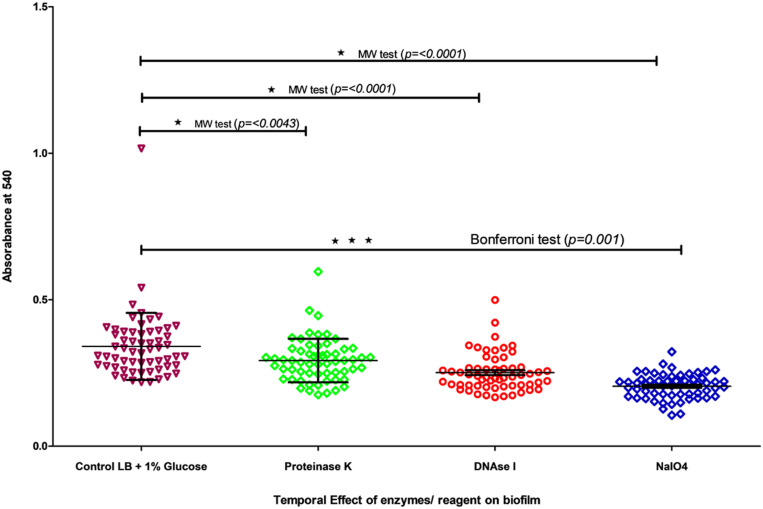
Temporal effect of DNase I, Proteinase K, and NaIO_4_ on biofilm was grown in LB for 24 h. The biofilm quantification was carried out by crystal violet assay at 540 nm. The treatment was carried out for 24 h. All dots/shapes represents strains and color represents different conditions of treatment. Color represent control and treatment with proteinase K, DNase I and NaIO_4_.

All 57 *V. cholerae* strains produced good biofilm with an OD_540_ value ranging from 0.22 to 1.01. These strains also a showed good percentage of detachment after treatment with (i) DNase I (Sigma, DN25), (ii) NaIO4 (Sigma, S1878), and (iii) proteinase K (Sigma, P2308). The anti-biofilm reagents were unable to degrade their respective analog component in matrix indicating that the biofilm matrix makes the reagents unable to penetrate the biofilm. However, at 0 h, the anti-biofilm agents degrade their respective components and prevent the biofilm formation indicating that these components play an important role in biofilm formation. Statistical analysis also showed that there was a significant difference between the non-treated and treated biofilm (Figure 1 and Supplementary Table S3) (Mann–Whitney test, *p* = 0.001). Analysis performed through one-way ANOVA (Bonferroni test) for DNase, NaIO_4_, and proteinase K-treated biofilm also showed a significant difference between control and treated samples (*p* < 0.001).

### MLST Analysis

Multilocus sequence typing analysis of *V. cholerae* strains showed that the number of polymorphic sites varies from 47 (*recA*) to 101 (*pgm*) (Supplementary Figures S1A–E). Of the five housekeeping genes, *asd*, amplicon size 543 bp, showed 71 polymorphic sites, 16 informative, 33 singletons, 57 synonymous, and 13 non-synonymous sites. The *dnaE*, amplicon size 502 bp, showed 83 polymorphic sites, 23 informative, 41 singletons, 65 synonymous, and 16 non-synonymous sites; whereas *lap*, amplicon size 476 bp, showed 69 polymorphic sites, nine informative, 19 singletons, 53 synonymous, and 14 non-synonymous sites. The *pgm*, amplicon size 553 bp showed 101 polymorphic sites, 65 informative, 23 singletons, 94 synonymous, and six non-synonymous sites. Similarly, *recA*, amplicon size 686 bp, showed 47 polymorphic sites, 36 informative, one singleton, 34 synonymous, and 10 non-synonymous sites (Table 2).

**FIGURE 2:**
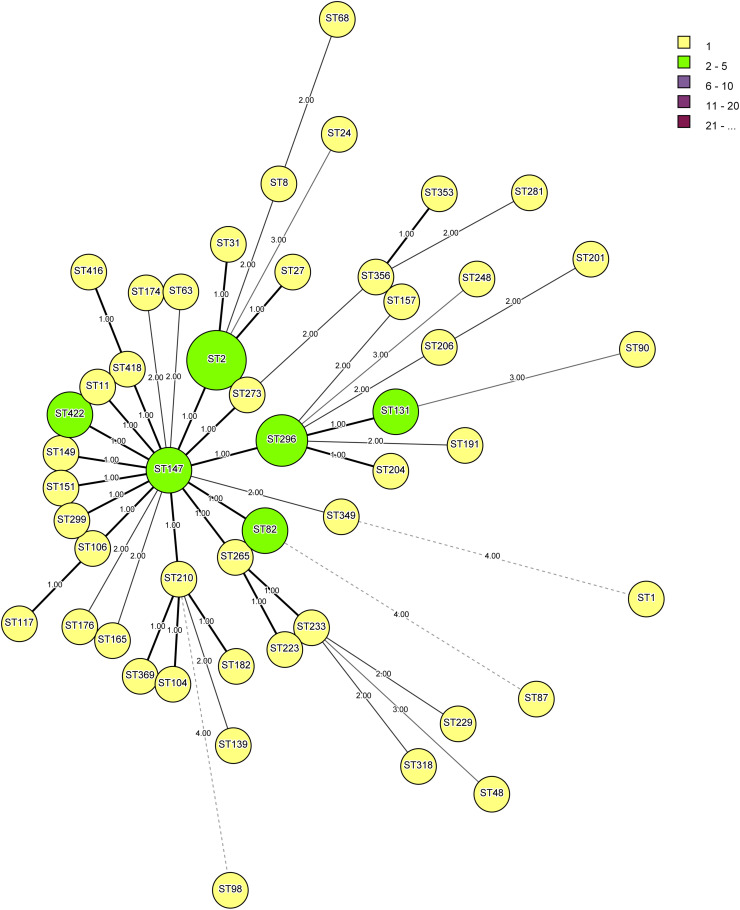
Minimum spanning tree is showing the relationship between different sequence types (STs) assigned by multilocus sequence typing (MLST) analysis of five housekeeping genes, namely, aspartate-semialdehyde dehydrogenase (*asd*), DNA polymerase III α-holoenzyme (*dnaE*), leucine aminopeptidase (*lap*), phosphoglucomutase (*pgm*), and recombinase repair protein A (*recA*). The minimum difference of three alleles separates the nodes into different clusters.

Analysis of 54 strains of *V. cholerae* O1, three strains of O139, and ATCC N16961 showed a total of 422 sequence types (STs) and formed eight clusters (Table 3). Strains DN34 and DN65 belonged to ST147 and showed a central clonal complex of an evolutionary tree. The primary cluster 1 consists of 42 STs, namely, ST2, ST8, ST11, ST27, ST31, ST63, ST68, ST82, ST104, ST106, ST117, ST139, ST149, ST157, ST165, ST174, ST176, ST182, ST191, ST201, ST304, ST206, ST210, ST223, ST229, ST233, ST265, ST151, ST281, ST299, ST318, ST147, ST349, ST296, ST273, ST353, ST356, ST369, ST131, ST422, ST418, and ST416 (Figure 2). The other seven clusters from two to eight represented only a single ST. For example, cluster 2 consists of ST 24, cluster 3 of ST248, cluster 4 of ST90, cluster 5 of ST48, cluster 6 of ST98, cluster 7 of ST87, and cluster 8 of ST1. The different groups were formed on the basis of a minimum difference in three alleles by separating the nodes. *V. cholerae* strains V1, AL40, 10, and VKO12 were clustered together and showed identical ST and belonged to ST2. This ST differed from central ST147 by a single allele, while DN47, DN69, and DN81 showed a similar ST296. Strains TV110 and TV123, DN34 and DN65, DN55 and DN73, and DN56 and DN64 were clustered together and showed STs belonged to ST82, ST147, ST131, and ST422 (Figure 2). Based on the above finding, we developed an MLST database for *V. cholerae* O1 and O139 and made it available at https://pubmlst.org/vcholerae/ to assign new STs among *V. cholerae* O1 and O139 strains.

**TABLE 3:** Allelic profiles of sequence types of *V. cholerae* O1 and O139 strains isolated from clinical and environmental sources.

**Sequence type**	**Number of isolates**	**Allelic Profile (Allele Number)**				
		***asd***	***dnaE***	***lap***	***Pgm***	***recA***
1	NI6961	1	1	1	1	1
2	V1, AL40, 10, VKO12	2	9	2	2	2
8	V20	2	9	2	12	4
11	DO183	2	2	2	16	2
24	32T	33	9	30	19	2
27	KO194	2	9	2	2	41
31	KO211	2	9	32	2	2
48	MO100	2	11	20	19	19
63	MO108	2	12	3	2	2
68	TV10	28	9	2	19	4
82	TV110, TV123	2	5	2	2	2
87	TV281	48	5	22	7	36
90	814B	51	2	15	77	19
98	IDH5/89	50	4	32	77	36
104	CH486	33	4	2	2	2
106	10T	2	3	2	2	2
117	RHV450	26	2	2	108	2
139	RHV16	55	4	2	2	19
149	2179	2	40	2	2	2
157	NICED1	2	46	2	104	4
165	NICED9	2	44	2	90	2
174	381	2	14	2	7	2
176	224	2	33	2	104	2
182	TVM149	2	4	2	98	2
191	TVM227	2	16	2	31	4
201	TVM152	2	15	2	64	38
304	TVM182	2	17	2	2	4
206	TVM261	2	15	2	11	4
210	TVM131	2	4	2	2	2
223	TVM134	16	51	2	2	2
229	TVM137	22	54	2	2	19
233	DN3	2	51	2	2	19
248	DN4	2	54	3	19	4
265	DN5	2	38	2	90	2
151	DN13	2	42	2	2	2
281	DN17	32	6	3	2	19
299	DN19	2	2	2	68	2
318	DN23	2	7	2	81	19
147	DN34, DN65	2	2	2	2	2
349	DN36	2	2	3	2	1
296	DN47, DN69, DN81	2	2	2	2	4
273	DN50	2	6	2	2	2
353	DN51	33	6	3	2	4
356	DN52	33	6	3	2	2
369	DN54	2	4	2	112	2
131	DN55, DN73	2	2	15	2	4
422	DN56, DN64	2	2	2	2	36
418	DN74	2	2	2	2	9
416	DN78	2	2	15	2	9

### PFGE

*Not*I-enzyme-digested genomic DNA of *V. cholerae* isolated from 1975 to 2015 from different parts of India produced a number of bands ranging from 22 to 24 and a molecular size from 25 to 700 kb (Supplementary Figure S2). Dendrogram generated from the PFGE fingerprint showed six fingerprint patterns namely E, F, G, H, I, and J among 33 strains. Strain N16961, carrying El Tor *ctxB* clustered with B representing the O139 strain was entirely different from other El Tor strains. Nineteen clinical strains from Silvassa isolated during 2012–2015 and carrying Haitian *ctxB* showed a fingerprint pattern classified as cluster A (A1–A10). Of the five clinical strains, two strains of O1 isolated from Silvassa and two clinical strains of O139 from Kerala that carried classical *ctxB* and one clinical strain from Dadra and Nagar Haveli that carried Haitian *ctxB* clustered together and formed cluster B (B1–B3). The *V. cholerae* 569B strain showed a closely related pattern D. The 10 O1 strains from Varanasi comprising seven clinical, two environmental, and one O1 clinical strain from Kerala showed a similar PFGE pattern F (F1–F10). However, El Tor strain N16961 showed a pattern related to this was designated as E. Three clinical strains of *V. cholerae* O1 from Kerala, two from Varanasi, one clinical strain from Delhi, and one reference strain of O139 MO10 from Madras showed a similar PFGE pattern and was designated as G (G1–G7). Similarly, 10 clinical strains of O1 from Kerala showed a closely related pattern H (H1–H10); however, another clinical strain of O139 from Kerala showed a distinct pattern and was designated as I (Supplementary Figure S2).

## Discussion

Cholera pandemics have been occurring in the world with the evolution of El Tor variants in the last 200 years (Rahaman et al., 2015). The first six pandemics were caused by the classical biotype; however, the seventh pandemic was caused by the El Tor biotype (Barua, 1992). In 1992, the O139 serogroup that was first isolated from diarrheal patient in Madras spread to other parts of India and Asian countries (Ramamurthy et al., 1993; Sinha et al., 2002). In the present study, all strains of *V. cholerae* O1, except five strains, showed a variation in phenotypic properties thus indicating the presence of variants of El Tor or a hybrid variant of El Tor biotypes. Three strains of O139 also showed a variation in phenotypic characters that carried either classical or El Tor *ctxB*/*rstR* genes. These strains also contained virulence-associated genes that were located on chromosome I, except for *hlyA* which was located on chromosome II. These observations thus indicate there are continuous changes in phenotypic characteristics in acquisition of the variant of the *ctxB* gene.

Hemolysin is another factor which was used to distinguish biotypes of *V. cholerae.* This factor cannot be used as a reliable marker for differentiation because all strains contain the hemolysin gene (*hlyA*^*class/El Tor*^) (Richardson et al., 1986; Singh et al., 2001; Raychoudhuri et al., 2008). These strains were also positive for *rtxA* and *rtxC* genes that can be used to discriminate the El Tor and classical biotypes (Chow et al., 2001). The presence of VSP-I and VC2346 in these strains confirmed that *V. cholerae* strains belonged to the seventh pandemic (Grim et al., 2010). The presence and integration of VSP-I in chromosome governed the evolution and rise of new strains.

The evolution of *V. cholerae* was determined by tracking the differences in the sequences of the *ctxB* gene (Kaper et al., 1995). It was reported that *V. cholerae* O1 strains possessed the characteristics of the El Tor biotype, carried classical *ctxB*, and were designated as altered El Tor strains (Evins et al., 1995; Nair et al., 2002). These strains caused a pandemic in Asian and African countries (Morita et al., 2008; Lizárraga-Partida et al., 2009); however, the Haitian *ctxB* carrying *V. cholerae* O1 was also reported from Asian and African countries (Goel et al., 2008; Naha et al., 2012; Bhattacharya et al., 2016; Das et al., 2016). *V. cholerae* O1 strains showing variations in phenotypic characters but carrying *rstA*^*El Tor*^, classical *ctxB*, or Haitian *ctxB* were designated as El Tor variants or Hybrid variants. These finding are similar to those who also reported the presence of mixed phenotype traits and carried either El Tor or classical *ctxB* and were designated as El Tor variants or hybrid variants (Raychoudhuri et al., 2009; Safa et al., 2010; Das et al., 2016). In this study, we proposed two variants, I and II, among O139 isolates.

All strains of *V. cholerae* produced good biofilm, and are developed on surface of substratum or at air liquid interface. These characteristics of air liquid interface biofilm formation indicated the rugosity of strains (Fong et al., 2010) and these properties had increased among the strain(s) with the respect to year of isolation. The detachment assay at initial treatment with inhibitory agents on biofilm significantly reduced biofilm formation and thus suggests the use of such anti-biofilm agents to prevent biofilm formation. Biofilm is another mechanism wherein it governs the tolerance against antibiotics and takes part in evolution by gene transfer (Savage et al., 2013). Biofilm is composed of extracellular polysaccharides making the penetration of antibiotics difficult (Lewenza, 2013). Also, it contained eDNA that can act as a source of transfer of antibiotic resistance or pathogenic genes from one cell to another and make them resistant or pathogenic (Savage et al., 2013). *V. cholerae* strains used in this study produced good biofilm, and the matrix is composed of eDNA, proteins, or polysaccharides.

Multilocus sequence typing based on number of housekeeping genes was used to study genomic diversity and general population structure (Maiden et al., 1998; Horwood et al., 2011; Luo et al., 2013). It is reproducible, but it is difficult to differentiate MLST schemes that utilize different housekeeping genes (Gonzalez-Escalona et al., 2008; Maiden et al., 2013). Therefore, in this study, we devised a new scheme for the MLST for *V. cholerae* O1 and O139 strains using five housekeeping genes *asd, dnaE, pgm, recA*, and *lap* and based on variation in the number of alleles present in each locus that ranges from 33 (*recA*) to 110 (*pgm*) with a mean of 5.17 alleles per locus. The finding of the present study thus suggests that the devised MLST scheme and developed database can be widely used to study the diversity and population structure of *V. cholerae* O1 and O139 strains. The developed database can select STs and designate new STs among *V. cholerae* after adding further sequence allele information to the database. The above MLST was reproducible that can be used for typing *V. cholerae* O1 and O139 strains. Another method, core genome multilocus sequence typing (cg-MLST), was introduced recently, which can provide good resolution, standardization, and ease of use for *V. cholerae* typing (Liang et al., 2020). It will require whole-genome sequencing data for generating MLST for *V. cholerae.* Although cg-MLST could be useful but need WGS data for analysis, MLST with five housekeeping genes can be used to type the *V. cholerae* strains.

PFGE was used for the investigation of the clonal relationship among the *V. cholerae* and other enteric pathogens (Arakawa et al., 2000; Vadivelu et al., 2000). However, in this study, no other enteric pathogens were used. *Not*I enzyme was used as a restriction fragment generating enzyme to study the genomic diversity of 57 *V. cholerae* strains isolated from different parts of India and from environmental and clinical sources. The PFGE pattern grouped 57 isolates, 54 O1 and three O139 strains, in three major pulsotypes on the basis of *ctxB* types and year of isolation. However, the isolates with El Tor *ctxB* were further grouped into six sub clusters differing with respect to sources and place of isolation. Moreover, the single Haitian *ctxB* strain grouped with classical *ctxB* carrying O1 strains. Although there is not much correlation between MLST and PFGE analysis, MLST is more discriminating than PFGE. These observations thus indicated that *V. cholerae* strains are undergoing continuous evolution with respect to pathogenic genes and genomic diversity.

## Data Availability Statement

All the sequences were submitted to GeneBank with accession numbers as MH169789 to MH169845 for *asd*, MH169846-MH169902 for *dnaE*, MH232265-MH232321 for *lap*, MH232322-MH232378 for *pgm*, and MH232379-MH232435 for *recA*.

## Ethics Statement

This study uses strains obtained from Clinical and Environmental Sources, and the Institutional Human Ethics Committee of the Institute of Life Sciences did not require the study to be reviewed or approved because the strains were from laboratory stock. Written informed consent for participation was not required for this study in accordance with the national legislation and the institutional requirements. The data were analyzed anonymously and reported.

## Author Contributions

AK and DS conceived and designed the experiments, analyzed the results, and wrote the manuscript. AK performed the experiments. Both authors reviewed and approved the manuscript.

## Conflict of Interest

The authors declare that the research was conducted in the absence of any commercial or financial relationships that could be construed as a potential conflict of interest.

## Footnotes

1 http://www.cdc.gov/pulsenet/PDF/vibrio_pfge_protocol-508c.pdf
