# Supplementary material for: Virulence Pattern and Genomic Diversity of Vibrio cholerae O1 and O139 Strains Isolated From Clinical and Environmental Sources in India
Source: Front Microbiol. 2020 Aug 26;11:1838. doi: 10.3389/fmicb.2020.01838 (PMC7479179; doi:10.3389/fmicb.2020.01838)

Supplementary Materials

Table S1: List of primers for virulence and regulatory genes, Vibrio seventh pathogenicity island and PCR conditions used in this study**.**

Primer	Target	Sequence	Size in bp	PCR Cyclic conditions	References
toxR-F	*toxR*	CCTTCGATCCCCTAAGCAATAC	779	ID: 94°C for 2 min<br>D: 94°C for 1 min<br>A: 62°C for 1 min } 20 cycles<br>E: 72 °C for 1 min<br>D: 94°C for 1 min<br>A: 54°C for 1 min } 10 cycles<br>E: 72 °C for 1 min<br>FE:72°C for 10 min	Singh et al. 2002
toxR-R		AGGGTTAGCAACGATGCGTAAG			
ace-F	*ace*	TAAGGATGTGCTTATGATGGACACCC	316		
ace-R		CGTGATGAATAAAGATACTCATAGG			
tcpA-F		CACGATAAGAAAACCGGTCAAGAG			
tcpA-class	*tcpA*^{class}	TTACCAAATGCAACGCCGAATG	620		
tcpA-El Tor	*tcpA*^{ET}	CGAAAGCACCTTCTTTCACACGTTG	453		
ctxA-F	*ctxA*	CGGGCAGATTCTAGACCTCCTG	564		
ctxA-R		CGATGATCTTGGAGCATTCCCAC			
ompU-F	*ompU*	ACGCTGACGGAATCAACCAAAG	869		
ompU-R		GCGGAAGTTTGGCTTGAAGTAG			
zot-F	*zot*	TCGCTTAACGATGGCGCGTTTT	947		
zot-R		AACCCCGTTTCACTTCTACCCA			
tcpI-F	*tcpI*	TAGCCTTAGTTCTCAGCAGGCA	862	D: 94°C for 1 min<br>A: 62°C for 1 min } 35 cycles<br>E: 72 °C for 1 min	Faruque et al. 1998
tcpI-R		GGCAATAGTGTCGAGCTCGTTA			
hylA ^{ET}F	*hylA*$^{class/El\ Tor}$	GGCAAACAGCGAAACAAATACC	481	ID: 94°C for 2 min<br>D: 94°C for 1 min<br>A: 62°C for 1 min } 30 cycles<br>E: 72 °C for 1 min<br>EF:72°C for 10 min	Rivera et al. 2001
hylA^{Cl}F		GAGCCGGCATTCATCTGAAT	727		
hylA-R		CTCAGCGGGCTAATACGGTTTA			

ctxB-F	*ctxB*	GATACACATAATAGAATTAAGGATG	449	ID: 94°C for 2 min	Mohapatra et al. 2011
ctxB-R		GGTTGCTTCTCATCATCGAACCCAC		D: 94°C for 1 min<br>A: 57°C for 30 sec } 35 cycles<br>E: 72 ˚C for 30 sec<br>FE:72°C for 10 min	
rstR^{class}-F	*rstR*^{class}	TCTCATCAGCAAAGCCTCCATC	243	ID: 94°C for 2 min	
rstR^{class}-R		GTAGCAAATGGTATCGGCGTTGG		D: 94°C for 30 sec	
rstR^{ET}-F	*rstR*$^{El\ Tor}$	GCACCATGATTTAAGATGCTC	320	A: 62°C for 30 sec } 35 cycles	
rstR^{ET}-R		GGCAATTAATAAGACTCAGGCAC		E: 72 ˚C for 30 sec<br>FE:72°C for 10 min	
CII-F	chromosomal location of CTXphage	CTCACGCTGAACAGCAAGTC	800	ID: 94°C for 2 min	Maiti et al. 2006.
CII-R		TTGCTTGAATCGAAAGGACA		D: 94°C for 1 min<br>A: 52°C for 1 min } 35 cycles<br>E: 72 ˚C for 1 min<br>FE:72°C for 10min	
ctxB-3	*ctxB*^{Haiti}	GTTTTACTATCTTCAGCATATGCGA	191	ID: 94°C for 2 min	Naha et al. 2012 (DMAMA-PCR)
Rv-cla		CCTGGTACTTCTACTTGAAACG		D: 94°C for 30 sec<br>A: 56°C for 30 sec } 30 cycles<br>E: 72 ˚C for 30 sec<br>FE:72°C for 10 min	
ctxB-4	*ctxB*^{class}	GTTTTACTATCTTCAGCATATGCGC	191	ID: 94°C for 2 min	
Rv-cla		CCTGGTACTTCTACTTGAAACG		D: 94°C for 30 sec<br>A: 60°C for 30 sec } 30 cycles<br>E: 72 ˚C for 30 sec<br>FE:72°C for 10 min	

Fw-com	*ctxB*$^{\text{El Tor}}$	ACTATCTTCAGCATATGCACATGG	254	ID: 94°C for 2 min<br>D: 94°C for 30 sec<br>A: 54°C for 30 sec — 30 cycles<br>E: 72 ˚C for 30 sec<br>FE:72°C for 10 min	Morita et al.2008<br>(MAMA-PCR)
Re-elt		CCTGGTACTTCTACTTGAAACA			
smp-F-<br>VC2346	*smp*	GCAACTGTGCTAGCAGTTGCCGTG	405	ID: 94°C for 2 min<br>D: 94°C for 1 min<br>A: 65°C for 30 sec — 30 cycles<br>E: 72 ˚C for 30 sec<br>FE:72°C for 10 min	Grim et al.2010
smp-R-<br>VC2346		GCCTGTTTCAAACGTGATGCGTA			
dcd-820F	*VC0175*	GCTTATTCAGC GCCCTCAGG TC	584	ID: 94°C for 2 min<br>D: 94°C for 1 min<br>A: 65°C for 30 sec — 30 cycles<br>E: 72 ˚C for 30 sec<br>FE:72°C for 10 min	
dcd-1403R		TAG CGT CGA GAT GAC ACA CCT TCG			
VSPI-F	VC0180-<br>VC0181	GCCGAGAACTCTAAAGCG CTTCTC	331	ID: 94°C for 2 min<br>D: 94°C for 1 min<br>A: 61°C for 30 sec — 30 cycles<br>E: 72 ˚C for 30 sec<br>FE:72°C for 10 min	
VSPI-R		CCAAGGTACAGATGAGTACCAGCA			
VC0174-F	Chr-I –VSPI<br>insertion	AAACTGGCGACCTTTGAGCAAGC	1321	ID: 94°C for 2 min<br>D: 94°C for 1 min<br>A: 65°C for 1 min — 30 cycles<br>E: 72 ˚C for 1 min<br>FE:72°C for 10 min	
VC0186-R		GATGGTAGCCTGACGCTGCATCTG			

VCA0695-F	Chr-II-VSPII insertion	ATAGCGGGAGTTGGCTCTGCA	957	ID: 94°C for 2 min<br>D: 94°C for 1 min<br>A: 65°C for 1 min } 30 cycles<br>E: 72 ˚C for 1 min<br>FE:72°C for 10 min	
VCA0697-R		GGTGACTTGGTGCCCATCGTA			

ID: Initial denaturation; D: Denaturation; A: Annealing; E: Extension; FE: Final extension.

References

Faruque, S. M., Asadulghani, Saha M. N., Alim A. R., Albert M. J., Islam K. M., et al. (1998). Analysis of clinical and environmental strains of nontoxigenic *Vibrio cholerae* for susceptibility to CTXPhi: molecular basis for origination of new strains with epidemic potential. *Infect. Immun.* 66(12)**,** 5819-5825. doi: 10.1128/IAI.66.12.5819-5825.1998

Grim, C.J., Choi, J., Chun, J., Jeon, Y.-S., Taviani, E., Hasan, N.A., et al. (2010). Occurrence of the *Vibrio cholerae* seventh pandemic VSP-I island and a new variant. *OMICS.* 14**,** 1-7. doi: 10.1089/omi.2009.0087

Maiti, D., Das, B., Saha, A., Nandy, R.K., Nair, G.B., and Bhadra, R.K. (2006). Genetic organization of pre-CTX and CTX prophages in the genome of an environmental *Vibrio cholerae* non-O1, non-O139 strain. *Microbiology.* 152**,** 3633-3641. doi: 10.1099/mic.0.2006/000117-0

Mohapatra S.S., Mantri C.K., Turabe Fazil M., Singh D.V. (2011) *Vibrio cholerae* O1 biotype El Tor strains isolated in 1992 from Varanasi, India harboured El Tor CTXΦ and classical ctxB on the chromosome-I and classical CTXΦ and classical *ctxB* on the chromosome-II. *Environ. Microbiol. Rep.* 3**,** 783-790. doi: 10.1111/j.1758-2229.2011.00287.x

Morita, M., Ohnishi, M., Arakawa, E., Bhuiyan, N., Nusrin, S., Alam, M., et al. (2008). Development and validation of a mismatch amplification mutation PCR assay to monitor the dissemination of an emerging variant of *Vibrio cholerae* O1 biotype El Tor. *Microbiol. Immunol.* 52**,** 314-317.doi: 10.1111/j.1348-0421.2008.00041.x

Naha, A., Pazhani, G.P., Ganguly, M., Ghosh, S., Ramamurthy, T., Nandy, R.K.. et al. (2012). Development and evaluation of a PCR assay for tracking the emergence and dissemination of Haitian variant ctxB in *Vibrio cholerae* O1 strains isolated from Kolkata, India. *J Clin Microbiol.* 50**,** 1733-1736.doi: 10.1128/JCM.00387-12

Rivera I.N., Chun J., Huq A., Sack R.B., Colwell R.R**.** (2001). Genotypes associated with virulence in environmental isolates of *Vibrio cholerae*. *Appl Environ Microbiol* **67:**2421-2429. doi: 10.1128/AEM.67.6.2421-2429.2001

Singh, D.V., Isac, S.R., and Colwell, R.R. (2002). Development of a hexaplex PCR assay for rapid detection of virulence and regulatory genes in *Vibrio cholerae* and *Vibrio mimicus*. *J Clin Microbiol.* 40**,** 4321-4324.doi: 10.1128/JCM.40.11.4321-4324.2002

Table S2. Multilocus sequence typing (MLST) primers for five housekeeping genes and PCR conditions used in this study.

Gene	Gene Product	Direction	Primer Sequence(s)	PCR cyclic conditions	References
dnaE	DNA polymerase III alpha subunit	Forward	CgRATMACCgCTTTCgCCg	ID: 98˚C for 30 sec<br>D: 98˚C for 10 sec<br>A: 50-52˚C for 30 sec } 35 cycles<br>E: 70˚C for 30 sec<br>FE:70˚C for 10 min	Garg et al. 2003
		Reverse	gAKATgTgTgAgCTgTTTgC		
lap	Leucine amino-peptidase	Forward	gAAgAggTCggTTTgCgAgg		
		Reverse	gTTTgAATggTgAgCggTTTgCT		
pgm	Phosphoglucomutase	Forward	CCKTCSCAYAACCCgCC		
		Reverse	TCRACRAACCATTTgAADCC		
recA	Recombination Repair Protein	Forward	gAAACCATTTCgACCggTTC		Kotetishvili et al. 2003
		Reverse	CCgTTATAgCTgTACCAAgCgCCC		
asd	Aspartate semialdehyde dehydrogenase	Forward	CgACTACgACATTCCTC		Karaolis et al. 1995
		Reverse	gTTATCCgCCCACTACCC		

ID: initial denaturation; D: denaturation; A: Annealing; E: extension; FE: final extension.

References

Garg, P., Aydanian, A., Smith, D., Morris J.G, Nair, G.B., and Stine, O.C. (2003). Molecular epidemiology of O139 *Vibrio cholerae*: mutation, lateral gene transfer, and founder flush. *Emerg. Infect. Dis.* 9**,** 810-814.doi: 10.3201/eid0907.020760

Kotetishvili, M., Stine, O.C., Chen, Y., Kreger, A., Sulakvelidze, A., Sozhamannan, S., et al. (2003). Multilocus sequence typing has better discriminatory ability for typing *Vibrio cholerae* than does pulsed-field gel electrophoresis and provides a measure of phylogenetic relatedness. *J. Clin. Microbiol.* 41**,** 2191-2196.doi: 10.1128/jcm.41.5.2191-2196.2003

Karaolis, D. K., Lan R. and Reeves, P.R. (1995). The sixth and seventh cholera pandemics are due to independent clones separately derived from environmental, nontoxigenic, non-O1 *Vibrio cholerae*. *J. Bacteriol.* 177(11)**,** 3191-3198. doi: 10.1128/jb.177.11.3191-3198.1995

Table S3. Temporal effect of DNase I, Proteinase K, and NaIO4on biofilm grown in Leuria Bertani Broth (Mann-Whitney test).

Sr. No	Treatment of Bio-film with different reagents	Mann-Whitney test (*p-value <0.05*)
1	Control Vs DNase I	< 0.0001
2	Control Vs Proteinase K	0.0043
3	Control Vs $NaIO_4$	<0.0001

Fig. S1a. Polymorphic sites of *asd* loci among *V. cholerae* isolates. The nucleotide designation was shown for allele one and for rest of alleles sites only polymorphism is shown along with synonymous and non-synonymous polymorphism.

asd

	1 1 1 1 1 1 1 1 1 2 2 2 2 2 2 2 2 3 3 3 3 3 3 3 3 3 3 4 4 4 4 4 4 4 4 4 4 4 4 4 4 5 5 5 5 5 5 5
	1 1 1 2 2 2 2 2 2 4 4 5 5 5 5 6 6 0 0 0 0 1 1 6 7 8 1 1 2 2 2 2 3 4 0 2 2 3 5 5 6 8 9 9 0 4 4 4 4 5 5 6 6 7 7 7 9 9 0 1 2 5 5 6 7
	1 4 5 6 7 8 6 7 8 1 2 3 4 6 8 0 7 1 3 4 6 1 4 6 7 8 9 3 8 5 3 8 8 9 1 2 4 8 0 6 2 3 8 6 2 3 5 5 7 9 7 3 4 5 6 5 9 8 9 4 5 6 3 7 3 8 3 7 9 4 3
asd_1	C A G C G A A T C G T A C G C A T C T A T A C C A G C T C T C G A T A T C C C T G T T T C C A A G C T A T G T C G G T T A A C T C G T A T T
asd_2	. . . . T T G A . A G A T . . . . . A . A G . . G . . . . G . . . A . . . G A . . . . . C A G A A
asd_6	. . . A . . . . . . C . . . . T T G A . A G A T . . . . . A . A G . . G . . . . G . . . A . . . G A . . . . . C A G A A
asd_7	. . . A C . . . . . C . . . . T T G A . A G A T . . . . . A . A G . . G . . . . G . . . A . . . G A . . . . . C A G A A
asd_8	. . . A C T . . . . C . . . . T T G A . A G A T . . . . . A . A G . . G . . . . G . . . A . . . G A . . . . . C A G A A
asd_9	G . . . . T T G A . A G A T . . . . . A . A G . . G . . . . G . . . A . . . G A . . . . . C A G A A
asd_10	. . . . T T G A . A G A T . . . . . A . A G . . G . . . . G . . . A . . . G A . . . . . C A G A A
asd_11	. . . . T T G A . A G A T . . . . . A . A G . . G . . . . G . . . A . . . G A . . . . . C A G A A
asd_13	. . . . T T G A . A G A T . . . . . A . A G A . G . . . . G . . . A . . . G A . . . . . C A G A A
asd_14	. . . . T T G A . A G A T . . . . . A . A G A C G . . . . G . . . A . . . G A . . . . . C A G A A
asd_15	. . . . T T G A . A G A T . . . . . A . A G A C G C . . . . . . G . . . A . . . G A . . . . . C A G A A
asd_16	. . . . T T G A . A G A T . . . . . A . A G A C G C A . . . . . G . . . A . . . G A . . . . . C A G A A
asd_17	. . . . T T G A . A G A T . . . . . A . A G A C G C A G . . . . G . . . A . . . G A . . . . . C A G A A
asd_18	. . . . T T G A . A G A T . . . . . A . A G A C G C A G T . . . G . . . A . . . G A . . . . . C A G A A
asd_19	. . . . T T G A . A G A T . . . . . A . A G A C G C A G T G . . G . . . A . . . G A . . . . . C A G A A
asd_20	. . . . T T G A . A G A T . . . . . A . A G A C G C A G T G A . G . A . A . . . G A . . . . . C A G A A
asd_21	. . . . T T G A . A G A T . . . . . A . A G A C G C A G T G A . G . A . A G . . G A . . . . . C A G A A
asd_22	. . . . T T G A . A G A T . . . . . A . A G A C G C A G T G A . G . A . A G T . G A . . . . . C A G A A
asd_23	. . . . T T G A . A G A T . . . . . A . A G A C G C A G T G A . G . A . A G T . G A . G . . . C A G A A
asd_24	. . . . T T G A . A G A T . . . . . A . A G A C G C A G T G A . G . A . A G T . G A . G . . . . A G A A
asd_26	T . . . . T T G A . A G A T . . . . . A . A G . . G . . . . G . . . A . . . G A . . . . . C A G A A
asd_28	T . . . . T . . . . T T G A . A G A T . . . . . A . A G . . G . . . . G . . . A . . . G A . . . . . C A G A A
asd_29	T . . . . T . G . . . . T T G A . A G A T . . . . . A . A G . . G . . . . G . . . A . . . G A . . . . . C A G A A
asd_30	T . . . . T . G T . . . . T T G A . A G A T . . . . . A . A G . . G . . . . G . . . A . . . G A . . . . . C A G A A
asd_31	T . . A . . . . T . . . . T T G A . A G A T . . . . . A . A G . . G . . . . G . . . A . . . G A . . . . . C A G A A
asd_32	T C . A . . . . T . . . . T T G A . A G A T . . . . . A . A G . . G . . . . G . . . A . . . G A . . . . . C A G A A
asd_33	T C A A . . . . T . . . . T T G A . A G A T . . . . . A . A G . . G . . . . G . . . A . . . G A . . . . . C A G A A
asd_34	T C A A . C . . T . . . . T T G A . A G A T . . . . . A . A G . . G . . . . G . . . A . . . G A . . . . . C A G A A
asd_35	T C A A . C . A T . . . . T T G A . A G A T . . . . . A . A G . . G . . . . G . . . A . . . G A . . . . . C A G A A
asd_36	T C A A . C . A T C . . . . T T G A . A G A T . . . . . A . A G . . G . . . . G . . . A . . . G A . . . . . C A G A A
asd_37	T C A A . C . A T C C . . . . T T G A . A G A T . . . . . A . A G . . G . . . . G . . . A . . . G A . . . . . C A G A A
asd_38	T C A A . C . A T C C . T . . . . T T G A . A G A T . . . . . A . A G . . G . . . . G . . . A . . . G A . . . . . C A G A A
asd_39	T C A A . C . A T C C . T T . . . . T T G A . A G A T . . . . . A . A G . . G . . . . G . . . A . . . G A . . . . . C A G A A
asd_40	T C A A . C . A T C C . T T T . . . . T T G A . A G A T . . . . . A . A G . . G . . . . G . . . A . . . G A . . . . . C A G A A
asd_41	T C A A . C . A T C C . T T T C . . . . . . T T G A . A G A T . . . . . A . A G . . G . . . . G . . . A . . . G A . . . . . C A G A A
asd_42	T C A A . C . A T C C . T T T C C . . . . . T T G A . A G A T . . . . . A . A G . . G . . . . G . . . A . . . G A . . . . . C A G A A
asd_43	T C A A . C . A T C C . T T T C C G . . . . T T G A . A G A T . . . . . A . A G . . G . . . . G . . . A . . . G A . . . . . C A G A A
asd_44	T C A A . C . A T C C . T T T C C G G . . . T T G A . A G A T . . . . . A . A G . . G . . . . G . . . A . . . G A . . . . . C A G A A
asd_45	T C A A . C . A T C C . T T T C C G G T . . T T G A . A G A T . . . . . A . A G . . G . . . . G . . . A . . . G A . . . . . C A G A A
asd_46	T C A A . C . A T C C . T T T C C G G T G . T T G A . A G A T . . . . . A . A G . . G . . . . G . . . A . . . G A . . . . . C A G A A
asd_47	T C A A . C . A T C C . T T T C C G G T G G T T G A . A G A T . . . . . A . A G . . G . . . . G . . . A . . . G A . . . . . C A G A A
asd_48	. . . . C . . . T T G A T A G A T . . . . . A . A G . . G . . . . G . . . A . . . G A . . . . . C A G A A
asd_49	. . . . C . . . T T G A T A G A T A . . . . A . A G . . G . . . . G . . . A . . . G A . . . . . C A G A A
asd_50	. . . . C . . . T T G A T A G A T A C . . . A . A G . . G . . . . G . . . A . . . G A . . . . . C A G A A
asd_51	. . . . C . . . T T G A T A G A T A C A . . A . A G . . G . . . . G . . . A . . . G A . . . . . C A G A A
asd_52	. . . . C . . . T T G A T A G A T A C A G . A . A G . . G . . . . G . . . A . . . G A . . . . . C A G A A
asd_53	. . . . C . . . T T G A T A G A T A C A G A A . A G . . G . . . . G . . . A . . . G A . . . . . C A G A A
asd_54	. . . . C . . . T T G A T A G A T A C A G A A T A G . . G . . . . G . . . A . . . G A . . . . . C A G A A
asd_55	. . . . C . . . T T G A T A G A T A C A G A A T A G . . G . . . . G . . . A . . . G A . . . . . C A G A A
asd_56	. . . . C . . . T T G A T A G A T A C A G A A T A G . . G . . . . . . T G . . . A . . . G A . . . . . C A G A A
asd_57	. . . . C . . . T T G A T A G A T A C A G A A T A G . . G . . . . . . T G . . . A . . . G A G . . . . C A G A A
asd_58	. . . . C . . . T T G A T A G A T A C A G A A T A G . . G . . . . . . T G . . . A . . . G A G . T . . C A G A A
asd_59	. . . . C . . . T T G A T A G A T A C A G A A T A G . . G . . . . . . T G . . . A . . . G A G . T G . C A G A A
asd_60	. . . . C . . . T T G A T A G A T A C A G A A T A G . . G . . . . . . T G . . . A . . . G A G . T G T C A G A A
asd_61	. . T . . . . C . . . T T G A . A G A T . . . . . A . A G . . G . . . . G . . . A . . . G A . . . . . C A G A A
asd_62	. . . G . . . . C . . . T T G A . A G A T . . . . . A . A G . . G . . . . G . . . A . . . G A . . . . . C A G A A
	S S S S S S S S S S S S S S S S S S S S S S N N S S S S N N S S S S S S N S S S S S S S S S S S S S N S S S N S S S N N S S S S S S N N N N

Fig. S1b. Polymorphic sites of *dnaE* loci among *V. cholerae* isolates. The nucleotide designation was shown for allele one and for rest of alleles sites only polymorphism is shown along with synonymous and non-synonymous polymorphism.

dnaE															1	1	1	1	1	1	1	1	1	1	2	2	2	2	2	2	2	2	3	3	3	3	3	3	3	3	3	3	3	3	3	3	3	3	3	3	3	3	3	3	3	3	3	4	4	4	4	4	4	4	4	4	4	4	4	4	4	4	4	4	4	4	4	4	4	4	4	4	5	5
		2	2	2	4	4	5	5	5	5	5	5	9	9	0	0	0	0	2	2	2	2	6	8	1	3	4	6	7	7	8	9	0	1	4	4	6	6	6	7	7	7	7	7	7	7	7	7	8	8	8	8	8	9	9	9	9	0	0	0	0	0	0	1	1	1	2	2	2	2	2	2	4	4	6	8	8	8	9	9	9	9	0	0
	1	1	8	9	4	5	0	1	3	4	5	6	1	5	0	1	2	6	6	7	8	9	7	8	8	2	7	9	0	1	4	7	7	9	4	7	4	7	9	0	1	2	3	4	5	6	7	9	4	5	6	7	9	0	1	2	9	2	4	5	6	8	9	0	1	5	2	3	4	5	6	7	0	7	4	5	6	8	0	1	2	7	1	2
Allele_1	G	G	G	T	C	T	T	C	A	G	A	G	C	G	G	T	C	A	G	C	C	A	C	C	T	T	T	C	G	C	C	C	C	C	A	T	C	C	C	A	T	C	C	C	G	C	C	G	G	A	A	T	T	G	T	C	A	T	C	G	C	C	A	A	T	C	C	G	T	T	G	C	C	T	T	C	A	C	A	T	A	A	C	
Allele_2	.	.	.	.	.	.	.	A	.	.	.	A	T	A	.	A	T	.	.	.	.	T	A	A	A	A	.	.	A	G	G	A	T	T	.	A	.	.	.	.	.	.	.	.	.	.	.	.	.	.	.	.	.	.	C	A	T	C	G	C	.	.	.	.	.	A	.	.	.	.	A	A	.	.	.	.	.	.	.	.	.	.	.	
Allele_3	.	.	.	A	.	.	.	A	.	.	.	A	T	A	.	A	T	.	.	.	.	T	A	A	A	A	.	.	A	G	G	A	T	T	.	A	.	.	.	.	.	.	.	.	.	.	.	.	.	.	.	.	.	.	C	A	T	C	G	C	.	.	.	.	.	A	.	.	.	.	A	A	.	.	.	.	.	.	.	.	.	.	.	
Allele_4	.	.	.	A	A	.	.	A	.	.	.	A	T	A	.	A	T	.	.	.	.	T	A	A	A	A	.	.	A	G	G	A	T	T	.	A	.	.	.	.	.	.	.	.	.	.	.	.	.	.	.	.	.	.	C	A	T	C	G	C	.	.	.	.	.	A	.	.	.	.	A	A	.	.	.	.	.	.	.	.	.	.	.	
Allele_5	.	.	.	A	A	A	.	A	.	.	.	A	T	A	.	A	T	.	.	.	.	T	A	A	A	A	.	.	A	G	G	A	T	T	.	A	.	.	.	.	.	.	.	.	.	.	.	.	.	.	.	.	.	.	C	A	T	C	G	C	.	.	.	.	.	A	.	.	.	.	A	A	.	.	.	.	.	.	.	.	.	.	.	
Allele_6	.	.	.	.	.	.	.	A	.	.	.	A	T	A	.	A	T	.	.	.	.	T	A	A	A	A	.	.	A	G	G	A	T	T	.	A	.	.	.	.	.	.	.	.	.	.	.	.	.	.	.	.	.	.	C	A	T	C	G	C	.	.	.	.	.	A	.	.	.	.	A	A	.	.	C	.	.	.	.	.	.	.	.	
Allele_7	C	.	.	.	.	.	.	A	.	.	.	A	T	A	.	A	T	.	.	.	.	T	A	A	A	A	.	.	A	G	G	A	T	T	.	A	.	.	.	.	.	.	.	.	.	.	.	.	.	.	.	.	.	.	C	A	T	C	G	C	.	.	.	.	.	A	.	.	.	.	A	A	.	.	.	.	.	.	.	.	.	.	.	
Allele_8	C	.	.	.	.	.	.	A	.	.	.	A	T	A	.	A	T	.	.	.	.	T	A	A	A	A	.	.	A	G	G	A	T	T	.	A	.	G	.	.	.	.	.	.	.	.	.	.	.	.	.	.	.	.	C	A	T	C	G	C	.	.	.	.	.	A	.	.	.	.	A	A	.	.	.	.	.	.	.	.	.	.	.	
Allele_9	T	.	.	.	.	.	.	A	.	.	.	A	T	A	.	A	T	.	.	.	.	T	A	A	A	A	.	.	A	G	G	A	T	T	.	A	.	.	.	.	.	.	.	.	.	.	.	.	.	.	.	.	.	.	C	A	T	C	G	C	.	.	.	.	.	A	.	.	.	.	A	A	.	.	.	.	.	.	.	.	.	.	.	
Allele_10	.	.	.	.	.	.	.	A	.	.	.	A	T	A	.	A	T	.	.	.	.	T	A	A	A	A	.	.	A	G	G	A	T	T	.	A	.	.	.	.	.	.	.	.	.	.	.	.	.	.	.	.	.	.	C	A	T	C	G	C	.	.	.	.	.	A	.	.	.	.	A	A	.	.	C	A	.	.	.	.	.	.	.	
Allele_11	.	.	.	.	.	.	.	A	.	.	.	A	T	A	.	A	T	.	.	.	.	T	A	A	A	A	.	.	A	G	G	A	T	T	.	A	T	.	.	.	.	.	.	.	.	.	.	.	.	.	.	.	.	.	C	A	T	C	G	C	.	.	.	.	.	A	.	.	.	.	A	A	.	.	.	.	.	.	.	.	.	.	.	
Allele_12	.	.	.	.	.	.	.	A	.	.	.	A	T	A	.	A	T	.	.	.	.	T	A	A	A	A	.	.	A	G	G	A	T	T	.	A	T	.	.	T	.	.	.	.	.	.	.	.	.	.	.	.	.	.	C	A	T	C	G	C	.	.	.	.	.	A	.	.	.	.	A	A	.	.	.	.	.	.	.	.	.	.	.	
Allele_14	.	.	.	.	.	.	.	A	.	.	.	A	T	A	.	A	T	.	.	.	.	T	A	A	A	A	.	.	A	G	G	A	T	T	.	A	T	.	.	T	.	.	.	.	.	.	.	.	.	.	.	.	.	.	C	A	T	C	G	C	.	.	.	T	.	A	.	.	.	.	A	A	.	.	.	T	.	.	.	.	.	.	.	
Allele_15	.	.	.	.	.	A	A	A	.	.	.	A	T	A	.	A	T	.	.	.	.	T	A	A	A	A	.	.	A	G	G	A	T	T	.	A	.	.	.	.	.	.	.	.	.	.	.	.	.	.	.	.	.	.	C	A	T	C	G	C	.	.	.	.	.	A	.	.	.	.	A	A	.	.	.	.	.	.	.	.	.	.	.	
Allele_16	.	.	.	.	.	A	A	A	G	.	.	A	T	A	.	A	T	T	.	.	.	T	A	A	A	A	.	.	A	G	G	A	T	T	.	A	.	.	.	.	.	.	.	.	.	.	.	.	.	.	.	.	.	.	C	A	T	C	G	C	.	.	.	.	.	A	.	.	.	.	A	A	.	.	.	.	.	.	.	.	.	.	.	
Allele_17	.	.	.	.	.	A	A	A	G	.	G	A	T	A	.	A	T	T	C	.	.	T	A	A	A	A	.	.	A	G	G	A	T	T	.	A	.	.	.	.	.	.	.	.	.	.	.	.	.	.	.	.	.	.	C	A	T	C	G	C	.	.	.	.	.	A	.	.	.	.	A	A	.	.	.	.	.	.	.	.	.	.	.	
Allele_18	.	.	.	.	.	A	A	A	G	.	G	A	T	A	.	A	T	T	C	T	.	T	A	A	A	A	.	.	A	G	G	A	T	T	.	A	.	.	.	.	.	.	.	.	.	.	.	.	.	.	.	.	.	.	C	A	T	C	G	C	.	.	.	.	.	A	.	.	.	.	A	A	.	.	.	.	.	.	.	.	.	.	.	
Allele_19	.	.	.	.	.	A	A	A	G	.	G	A	T	A	.	A	T	T	C	T	T	T	A	A	A	A	.	.	A	G	G	A	T	T	.	A	.	.	.	.	.	.	.	.	.	.	.	.	.	.	.	.	.	.	C	A	T	C	G	C	.	.	.	.	.	A	.	.	.	.	A	A	.	.	.	.	.	.	.	.	.	.	.	
Allele_22	.	.	.	.	.	A	A	A	G	.	G	A	T	A	.	A	T	T	C	T	T	T	A	A	A	A	.	.	A	G	G	A	T	T	.	A	.	.	.	.	.	.	.	.	.	.	T	.	.	.	.	.	.	.	C	A	T	C	G	C	.	.	.	.	.	A	.	.	.	.	A	A	.	.	.	.	.	.	.	.	.	.	.	
Allele_23	.	.	.	.	.	A	A	A	.	.	.	A	T	A	.	A	T	.	.	.	.	T	A	A	A	A	.	.	A	G	G	A	T	T	.	A	.	.	.	.	C	.	.	.	.	.	.	.	.	.	.	.	.	.	C	A	T	C	G	C	.	.	.	.	.	A	.	.	.	.	A	A	.	.	.	.	.	.	.	.	.	.	.	
Allele_24	.	.	.	.	.	A	A	A	.	.	.	A	T	A	.	A	T	.	.	.	.	T	A	A	A	A	.	.	A	G	G	A	T	T	.	A	.	.	.	.	C	.	.	.	.	.	.	.	.	.	.	.	.	.	C	A	T	C	G	T	.	.	.	.	.	A	.	.	.	.	A	A	.	.	.	.	.	.	.	.	.	.	.	
Allele_25	.	.	.	.	.	A	A	A	.	.	.	A	T	A	.	A	T	.	.	.	.	T	A	A	A	A	.	.	A	G	G	A	T	T	.	A	.	.	.	.	C	.	.	.	.	.	.	.	.	.	.	.	.	.	C	A	T	C	G	T	T	.	.	.	.	A	.	.	.	.	A	A	.	.	.	.	.	.	.	.	.	.	.	
Allele_26	.	.	.	.	.	A	A	A	.	.	.	A	T	A	.	A	T	.	.	.	.	T	A	A	A	A	.	.	A	G	G	A	T	T	.	A	.	.	.	.	C	.	.	.	.	.	.	.	.	.	.	.	.	.	C	A	T	C	G	T	T	T	.	.	.	A	.	.	.	.	A	A	.	.	.	.	.	.	.	.	.	.	.	
Allele_27	.	.	.	.	.	.	A	A	.	.	.	A	T	A	.	A	T	C	.	T	.	T	A	A	A	A	.	.	A	G	G	A	T	T	.	A	.	.	T	.	.	.	.	.	.	.	.	.	.	.	.	.	.	.	C	A	T	C	G	C	.	.	.	.	.	A	.	.	.	.	A	A	.	.	.	.	.	.	.	.	.	.	.	
Allele_28	.	.	.	.	.	.	A	A	.	.	.	A	T	A	.	A	T	C	T	T	.	T	A	A	A	A	.	.	A	G	G	A	T	T	.	A	.	.	T	.	.	.	T	.	.	.	.	.	.	.	.	.	.	.	C	A	T	C	G	C	.	.	.	.	.	A	.	.	.	.	A	A	.	.	.	.	.	.	.	.	.	.	.	
Allele_29	.	.	.	.	.	.	A	A	.	.	.	A	T	A	.	A	T	C	T	T	.	T	A	A	A	A	.	.	A	G	G	A	T	T	.	A	.	.	T	.	.	.	T	.	T	.	.	.	.	.	.	.	.	.	C	A	T	C	G	C	.	.	.	.	.	A	.	.	.	.	A	A	.	.	.	.	.	.	.	.	.	.	.	
Allele_30	.	.	.	.	.	.	A	A	.	.	.	A	T	A	.	A	T	C	T	T	.	T	A	A	A	A	.	.	A	G	G	A	T	T	.	A	.	.	T	.	.	.	T	.	T	.	T	.	.	.	.	.	.	.	C	A	T	C	G	C	.	.	.	.	.	A	.	.	.	.	A	A	.	.	.	.	.	.	.	.	.	.	.	
Allele_31	.	.	.	.	.	.	A	A	.	.	.	A	T	A	.	A	T	C	T	T	.	T	A	A	A	A	.	.	A	G	G	A	T	T	.	A	.	.	T	.	.	.	T	.	T	.	T	.	.	G	.	.	.	.	C	A	T	C	G	C	.	.	.	.	.	A	.	.	.	.	A	A	.	.	.	.	.	.	.	.	.	.	.	
Allele_32	.	.	.	.	.	.	A	A	.	.	.	A	T	A	.	A	T	C	T	T	.	T	A	A	A	A	.	.	A	G	G	A	T	T	.	A	.	.	T	.	.	.	T	.	T	.	T	.	.	G	.	G	.	.	C	A	T	C	G	C	.	.	.	.	.	A	.	.	.	.	A	A	.	.	.	.	.	.	.	.	.	.	.	
Allele_33	.	.	.	.	.	.	A	A	.	.	.	A	T	A	.	A	T	C	T	T	.	T	A	A	A	A	.	.	A	G	G	A	T	T	.	A	.	.	T	.	.	.	T	.	T	.	T	.	.	G	.	G	.	.	C	A	T	C	G	C	.	.	.	.	A	A	.	.	.	.	A	A	.	.	.	.	.	.	.	.	.	.	.	
Allele_35	.	.	.	.	.	A	A	A	.	.	.	A	T	A	.	A	T	.	T	.	.	T	A	A	A	A	.	.	A	G	G	A	T	T	.	A	.	.	.	.	C	.	.	.	.	.	.	.	.	.	.	.	.	.	C	A	T	C	G	C	.	.	.	.	.	A	.	.	.	.	A	A	.	.	.	.	.	.	.	.	.	.	.	
Allele_36	.	.	.	.	.	A	A	A	.	.	.	A	T	A	.	A	T	.	T	.	.	T	A	A	A	A	.	.	A	G	G	A	T	T	.	A	.	T	.	.	C	.	.	.	.	.	.	.	.	.	.	.	.	.	C	A	T	C	.	C	.	.	.	.	.	A	.	.	.	.	A	A	.	.	.	.	.	.	.	.	.	.	.	
Allele_38	.	.	.	.	.	A	A	A	.	.	.	A	T	A	.	A	T	.	T	.	.	T	A	A	A	A	.	.	A	G	G	A	T	T	.	A	.	.	.	T	.	.	.	G	.	.	.	.	.	.	.	.	.	.	C	A	T	C	G	C	.	.	.	.	.	A	.	.	.	.	A	A	.	.	.	.	.	.	.	.	.	.	.	
Allele_39	.	.	.	.	.	A	A	A	.	.	.	A	T	A	.	A	T	.	T	.	.	T	A	A	A	A	.	.	A	G	G	A	T	T	.	A	.	.	.	T	.	.	.	G	.	T	.	.	.	.	.	.	.	.	C	A	T	C	G	C	.	.	.	.	.	A	.	.	.	.	A	A	.	.	.	.	.	.	.	.	.	.	.	
Allele_40	.	A	.	.	.	.	.	A	.	.	.	A	T	A	.	A	T	.	.	.	.	T	A	A	A	A	.	.	A	G	G	A	T	T	.	A	.	.	.	.	.	.	.	.	.	.	.	.	.	.	.	.	.	.	C	A	T	C	G	C	.	.	.	.	.	A	.	.	.	.	A	A	.	.	.	.	.	.	.	.	.	.	.	
Allele_41	.	A	A	.	.	.	.	A	.	.	.	A	T	A	.	A	T	.	.	.	.	T	A	A	A	A	G	.	A	G	G	A	T	T	.	A	.	.	.	.	.	.	.	.	.	.	.	.	.	.	.	.	.	.	C	A	T	C	G	C	.	.	.	.	.	A	.	.	.	.	A	A	.	.	.	.	.	.	.	.	.	.	.	
Allele_42	.	A	A	.	.	.	.	A	.	.	.	A	T	A	.	A	T	.	.	.	.	T	A	A	A	A	G	G	A	G	G	A	T	T	.	A	.	.	.	.	.	.	.	.	.	.	.	.	.	.	.	.	.	.	C	A	T	C	G	C	.	.	.	.	.	A	.	.	.	.	A	A	.	.	.	.	.	.	.	.	.	.	.	
Allele_43	.	A	A	.	.	.	.	A	.	.	.	A	T	A	.	A	T	.	.	.	.	T	A	A	A	A	G	G	A	G	G	A	T	T	T	A	.	.	.	.	.	.	.	.	.	.	.	.	.	.	.	.	.	.	C	A	T	C	G	C	.	.	.	.	.	A	.	.	.	.	A	A	.	.	.	.	.	.	.	.	.	.	.	
Allele_44	.	A	A	.	.	.	.	A	.	.	.	A	T	A	.	A	T	.	.	.	.	T	A	A	A	A	G	G	A	G	G	A	T	T	T	A	.	.	.	.	.	.	.	.	.	.	.	C	.	.	.	.	.	.	C	A	T	C	G	C	.	.	.	.	.	A	.	.	.	.	A	A	.	.	.	.	.	.	.	.	.	.	.	
Allele_45	.	A	A	.	.	.	.	A	.	.	.	A	T	A	.	A	T	.	.	.	.	T	A	A	A	A	G	G	A	G	G	A	T	T	T	A	.	.	.	.	.	.	.	.	.	.	.	C	C	.	.	.	.	.	C	A	T	C	G	C	.	.	.	.	.	A	.	.	.	.	A	A	.	.	.	.	.	.	.	.	.	.	.	
Allele_46	.	A	A	.	.	.	.	A	.	.	.	A	T	A	.	A	T	.	.	.	.	T	A	A	A	A	G	G	A	G	G	A	T	T	T	A	.	.	.	.	.	.	.	.	.	.	.	C	C	C	.	.	.	.	C	A	T	C	G	C	.	.	.	.	.	A	.	.	.	.	A	A	.	.	.	.	.	.	.	.	.	.	.	
Allele_47	.	A	A	.	.	.	.	A	.	.	.	A	T	A	.	A	T	.	.	.	.	T	A	A	A	A	G	G	A	G	G	A	T	T	T	A	.	.	.	.	.	.	.	.	.	.	.	C	C	C	C	.	.	.	C	A	T	C	G	C	.	.	.	.	.	A	.	.	.	.	A	A	.	.	.	.	.	.	.	.	.	.	.	
Allele_49	.	A	A	.	.	.	.	A	.	.	.	A	T	A	.	A	T	.	.	.	.	T	A	A	A	A	G	G	A	G	G	A	T	T	T	A	.	.	.	.	.	.	.	.	.	.	.	C	C	C	C	.	.	T	C	A	T	C	G	C	.	.	.	.	.	A	.	.	.	.	A	A	.	.	.	.	.	.	.	.	.	.	.	
Allele_50	.	A	A	.	.	.	.	A	.	.	.	A	T	A	.	A	T	.	.	.	.	T	A	A	A	A	G	G	A	G	G	A	T	T	T	A	.	.	.	.	.	.	.	.	.	.	.	C	C	C	C	.	.	T	C	A	T	C	G	C	.	.	.	.	.	A	.	.	.	.	A	A	A	.	.	.	.	.	.	.	.	.	.	
Allele_51	.	A	A	.	.	.	.	A	.	.	.	A	T	A	.	A	T	.	.	.	.	T	A	A	A	A	G	G	A	G	G	A	T	T	T	A	.	.	.	.	.	.	.	.	.	.	.	C	C	C	C	.	.	T	C	A	T	C	G	C	.	.	.	.	.	A	.	.	.	.	A	A	A	A	.	.	.	.	.	.	.	.	.	
Allele_53	.	.	.	.	.	.	.	A	.	.	.	A	T	A	.	A	T	.	.	.	.	T	A	A	A	A	.	.	A	G	G	A	T	T	.	A	.	.	.	.	.	.	.	.	.	.	.	.	.	.	.	.	.	A	C	A	T	C	G	C	.	.	.	.	.	A	.	.	.	.	A	A	.	.	.	.	.	.	.	.	.	.	.	
Allele_54	.	.	.	.	.	.	.	A	.	.	.	A	T	A	.	A	T	.	.	.	.	T	A	A	A	A	.	.	A	G	G	A	T	T	.	A	.	.	.	.	.	.	.	.	.	.	.	.	.	.	.	.	.	A	C	A	T	C	G	C	.	.	.	.	.	A	.	.	.	.	A	A	.	.	.	.	C	.	.	.	.	.	.	
Allele_55	.	.	.	.	.	.	.	A	.	.	.	A	T	A	.	A	T	.	.	.	.	T	A	A	A	A	.	.	A	G	G	A	T	T	.	A	.	.	.	.	.	.	.	.	.	.	.	.	.	.	.	.	.	A	C	A	T	C	G	C	.	.	.	.	.	A	.	.	.	.	A	A	.	.	.	.	C	A	.	.	.	.	.	
Allele_56	.	.	.	.	.	.	.	A	.	.	.	A	T	A	.	A	T	.	.	.	.	T	A	A	A	A	.	.	A	G	G	A	T	T	.	A	.	.	.	.	.	.	.	.	.	.	.	.	.	.	.	.	.	A	C	A	T	C	G	C	.	.	.	.	.	A	.	.	.	.	A	A	.	.	.	.	C	A	C	.	.	.	.	
Allele_57	.	.	.	.	.	.	.	A	.	.	.	A	T	A	.	A	T	.	.	.	.	T	A	A	A	A	.	.	A	G	G	A	T	T	.	A	.	.	.	.	.	.	.	.	.	.	.	.	.	.	.	.	.	A	C	A	T	C	G	C	.	.	.	.	.	A	.	.	.	.	A	A	.	.	.	.	C	A	C	A	.	.	.	
Allele_58	.	.	.	.	.	.	.	A	.	.	.	A	T	A	.	A	T	.	.	.	.	T	A	A	A	A	.	.	A	G	G	A	T	T	.	A	.	.	.	.	.	.	.	.	.	.	.	.	.	.	.	.	.	A	C	A	T	C	G	C	.	.	.	.	.	A	.	.	.	.	A	A	.	.	.	.	C	A	C	A	C	.	.	
Allele_59	.	.	.	.	.	.	.	A	.	.	.	A	T	A	.	A	T	.	.	.	.	T	A	A	A	A	.	.	A	G	G	A	T	T	.	A	.	.	.	.	.	.	.	.	.	.	.	.	.	.	.	.	.	A	C	A	T	C	G	C	.	.	.	.	.	A	.	.	.	.	A	A	.	.	.	.	C	A	C	A	C	.	A	
Allele_60	.	.	.	.	.	.	.	A	.	.	.	A	T	A	.	A	T	.	.	.	.	T	A	A	A	A	.	.	A	G	G	A	T	T	.	A	.	.	.	.	.	.	.	.	.	.	.	.	.	.	.	.	.	A	C	A	T	C	G	C	.	.	.	.	.	A	.	.	.	.	A	A	.	.	.	.	C	A	C	A	C	.	A	
	S	N	S	S	S	S	S	N	S	S	S	S	N	N	S	S	N	S	S	S	S	N	N	N	N	N	S	S	S	N	N	S	N	S	S	N	S	S	S	S	S	S	S	S	S	S	S	S	S	S	S	S	S	S	S	N	S	S	S	S	S	S	S	S	S	N	S	S	S	S	S	S	S	S	S	S	N	S	S	S	S	S	N	

Fig. S1c. Polymorphic sites of *lap* loci among *V. cholerae* isolates. The nucleotide designation was shown for allele one and for rest of alleles sites only polymorphism is shown along with synonymous and non-synonymous polymorphism.

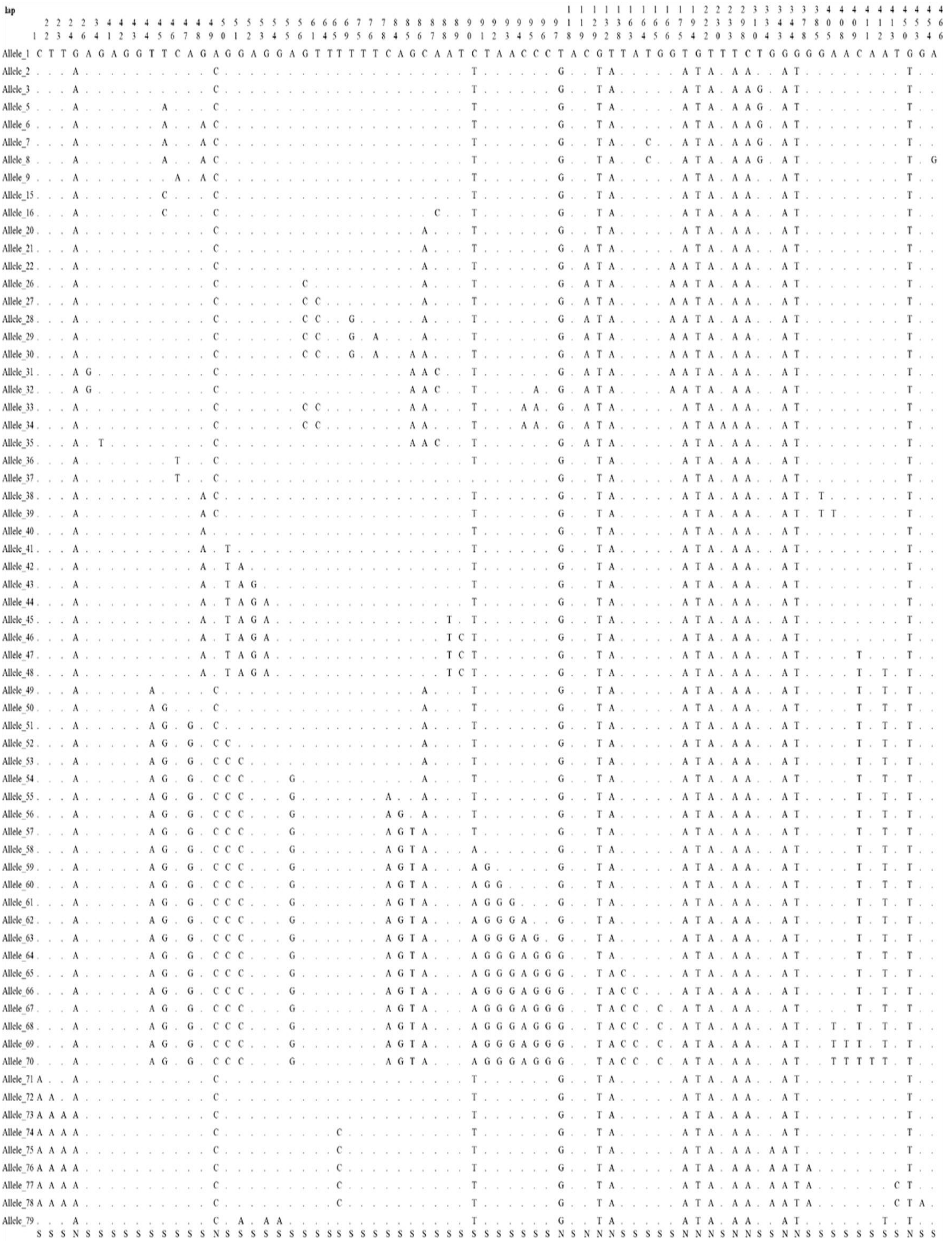

Fig. S1d. Polymorphic sites of *pgm* loci among *V. cholerae* isolates. The nucleotide designation was shown for allele one and for rest of alleles sites only polymorphism is shown along with synonymous and non-synonymous polymorphism.

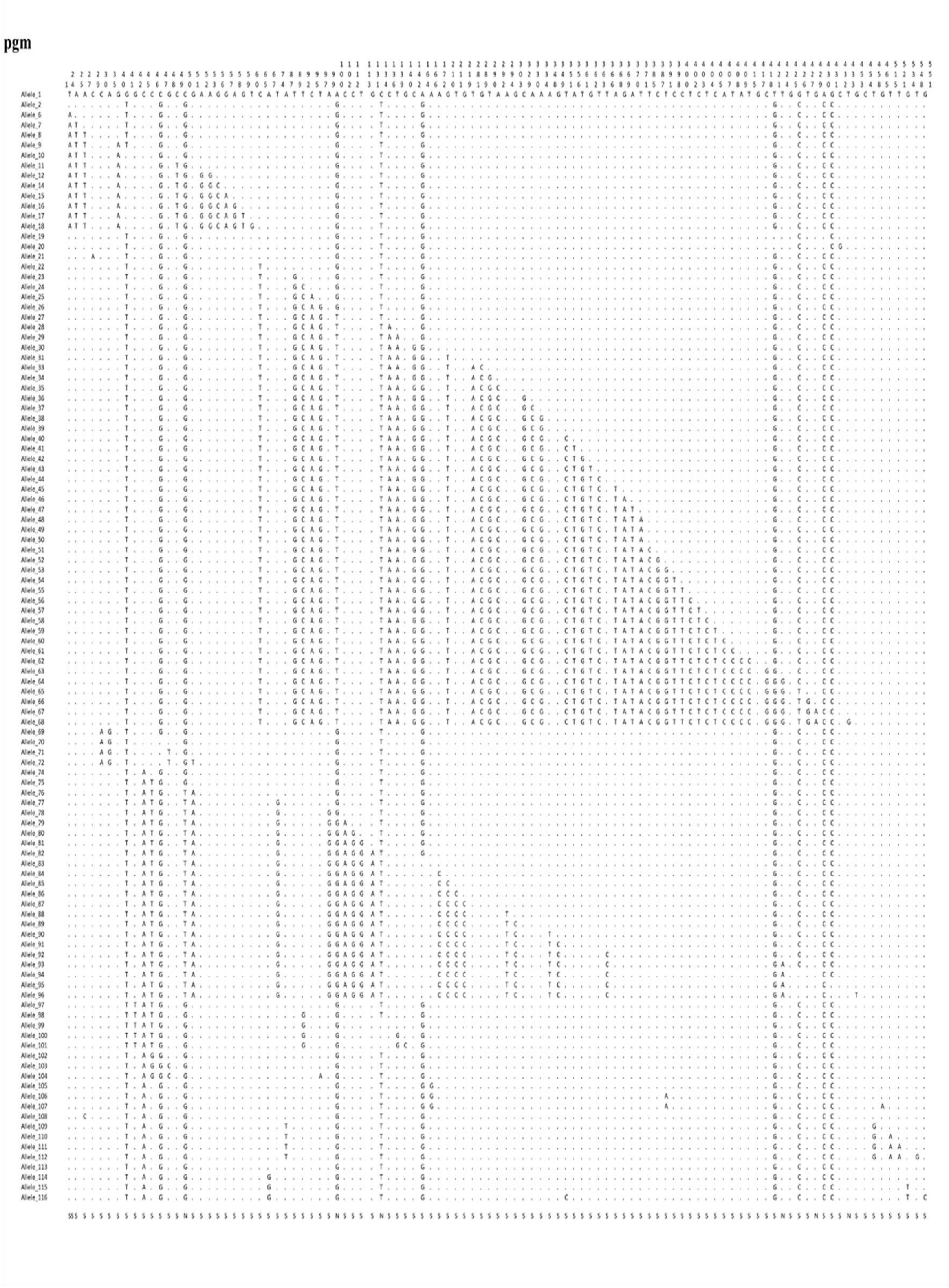

Fig. S1e. Polymorphic sites of *recA* loci among *V. cholerae* isolates. The nucleotide designation was shown for allele one and for rest of alleles sites only polymorphism is shown along with synonymous and non-synonymous polymorphism.

recA

	1	3	5	6	29	44	54	90	98	102	135	136	156	179	193	198	230	252	255	261	264	265	279	287	306	325	339	363	369	383	433	465	479	554	555	558	597	600	608	615	633	640	648	651	655	659	686
Allele_1	T	G	T	G	T	A	A	G	A	A	C	A	G	A	A	T	T	G	C	C	G	A	G	G	T	G	A	A	T	A	T	T	T	G	A	T	G	T	G	T	G	T	C	G	G	A	
Allele_2	G	A	.	A	C	T	.	A	T	.	.	.	A	.	.	A	.	.	.	.	.	.	T	T	A	.	.	T	A	T	.	A	G	.	T	.	.	A	.	.	.	.	T	.	A	.	
Allele_4	G	A	A	A	C	T	.	A	T	.	.	.	A	.	.	A	.	.	.	.	.	.	T	T	A	.	.	T	A	T	.	A	G	.	T	.	.	A	.	.	.	.	T	.	A	.	
Allele_5	G	A	A	A	C	T	G	A	T	.	.	.	A	.	.	A	.	.	.	.	.	.	T	T	A	.	.	T	A	T	.	A	G	.	T	.	.	A	.	.	.	.	T	.	A	.	
Allele_6	G	A	A	A	C	T	G	A	T	G	.	.	A	.	.	A	.	.	.	.	.	.	T	T	A	.	.	T	A	T	.	A	G	.	T	.	.	A	.	.	.	.	T	.	A	.	
Allele_7	G	A	A	A	C	T	G	A	T	G	T	.	A	.	.	A	.	.	.	.	.	.	T	T	A	.	.	T	A	T	.	A	G	.	T	.	.	A	.	.	.	.	T	.	A	.	
Allele_8	G	A	A	A	C	T	G	A	T	G	T	G	A	.	.	A	.	.	.	.	.	.	T	T	A	.	.	T	A	T	.	A	G	.	T	.	.	A	.	.	.	.	T	.	A	.	
Allele_9	G	A	A	A	C	T	G	A	T	G	T	G	A	G	.	A	.	.	.	.	.	.	T	T	A	.	.	T	A	T	.	A	G	.	T	.	.	A	.	.	.	.	T	.	A	.	
Allele_10	G	A	A	A	C	T	G	A	T	G	T	G	A	G	T	A	.	.	.	.	.	.	T	T	A	.	.	T	A	T	.	A	G	.	T	.	.	A	.	.	.	.	T	.	A	.	
Allele_11	G	A	A	A	C	T	G	A	T	G	T	G	A	G	T	A	G	.	.	.	.	.	T	T	A	.	.	T	A	T	.	A	G	.	T	.	.	A	.	.	.	.	T	.	A	.	
Allele_12	G	A	A	A	C	T	G	A	T	G	T	G	A	G	T	A	G	A	.	.	.	.	T	T	A	.	.	T	A	T	.	A	G	.	T	.	.	A	.	.	.	.	T	.	A	.	
Allele_13	G	A	A	A	C	T	G	A	T	G	T	G	A	G	T	A	G	A	G	.	.	.	T	T	A	.	.	T	A	T	.	A	G	.	T	.	.	A	.	.	.	.	T	.	A	.	
Allele_14	G	A	A	A	C	T	G	A	T	G	T	G	A	G	T	A	G	A	G	T	.	.	T	T	A	.	.	T	A	T	.	A	G	.	T	.	.	A	.	.	.	.	T	.	A	.	
Allele_15	G	A	A	A	C	T	G	A	T	G	T	G	A	G	T	A	G	A	G	T	.	C	T	T	A	.	.	T	A	T	.	A	G	.	T	.	.	A	.	.	.	.	T	.	A	.	
Allele_16	G	A	A	A	C	T	G	A	T	G	T	G	A	G	T	A	G	A	G	T	.	C	T	C	A	.	.	T	A	T	.	A	G	.	T	.	.	A	.	.	.	.	T	.	A	.	
Allele_17	G	A	A	A	C	T	G	A	T	G	T	G	A	G	T	A	G	A	G	T	.	C	T	C	A	T	.	T	A	T	.	A	G	.	T	.	.	A	.	.	.	.	T	.	A	.	
Allele_18	G	A	A	A	C	T	G	A	T	G	T	G	A	G	T	A	G	A	G	T	.	C	T	C	A	T	G	T	A	T	.	A	G	.	T	.	.	A	.	.	.	.	T	.	A	.	
Allele_19	G	A	A	A	C	T	G	A	T	G	T	G	A	G	T	A	G	A	G	T	.	C	T	C	A	T	G	T	A	T	C	A	G	.	T	.	.	A	.	.	.	.	T	.	A	.	
Allele_21	G	A	A	A	C	T	G	A	T	G	T	G	A	G	T	A	G	A	G	T	.	C	T	C	A	T	G	T	A	T	C	A	G	T	T	.	.	A	.	.	.	.	T	.	A	.	
Allele_22	G	A	A	A	C	T	G	A	T	G	T	G	A	G	T	A	G	A	G	T	.	C	T	C	A	T	G	T	A	T	C	A	G	T	T	G	.	A	.	.	.	.	T	.	A	.	
Allele_23	G	A	A	A	C	T	G	A	T	G	T	G	A	G	T	A	G	A	G	T	.	C	T	C	A	T	G	T	A	T	C	A	G	T	T	G	A	A	.	.	.	.	T	.	A	.	
Allele_24	G	A	A	A	C	T	G	A	T	G	T	G	A	G	T	A	G	A	G	T	.	C	T	C	A	T	G	T	A	T	C	A	G	T	T	G	A	A	C	.	.	.	T	.	A	.	
Allele_25	G	A	A	A	C	T	G	A	T	G	T	G	A	G	T	A	G	A	G	T	.	C	T	C	A	T	G	T	A	T	C	A	G	T	T	G	A	A	C	C	.	.	T	.	A	.	
Allele_26	G	A	A	A	C	T	G	A	T	G	T	G	A	G	T	A	G	A	G	T	.	C	T	C	A	T	G	T	A	T	C	A	G	T	T	G	A	A	C	C	.	C	T	.	A	.	
Allele_27	G	A	A	A	C	T	G	A	T	G	T	G	A	G	T	A	G	A	G	T	.	C	T	C	A	T	G	T	A	T	C	A	G	T	T	G	A	A	C	C	.	C	T	T	A	.	
Allele_28	G	A	A	A	C	T	G	A	T	G	T	G	A	G	T	A	G	A	G	T	.	C	T	C	A	T	G	T	A	T	C	A	G	T	T	G	A	A	C	C	.	C	T	T	A	G	
Allele_35	G	A	.	A	C	T	.	A	T	.	.	.	A	.	.	A	.	.	.	.	.	.	T	T	A	.	.	T	A	T	.	A	G	.	T	.	.	A	.	.	.	.	T	.	A	.	
Allele_36	G	C	.	A	C	T	.	A	T	.	.	.	A	.	.	A	.	.	.	.	.	.	T	T	A	.	.	T	A	T	.	A	G	.	T	.	.	A	.	.	.	.	T	.	A	.	
Allele_38	G	A	.	A	G	T	.	A	T	.	.	.	A	.	.	A	.	.	.	.	.	.	T	T	A	.	.	T	A	T	.	A	G	.	T	.	.	A	.	.	.	.	T	.	A	.	
Allele_39	G	A	.	A	G	T	.	A	T	.	.	.	A	.	.	A	.	.	.	.	.	.	T	T	A	.	.	T	A	T	.	A	G	.	T	.	.	A	.	.	.	.	T	.	A	.	
Allele_41	G	A	.	A	C	T	.	A	T	.	.	.	A	.	.	A	.	.	.	.	.	.	T	T	A	.	.	T	A	T	.	A	G	.	T	.	.	A	.	.	.	.	T	.	A	.	
Allele_42	G	A	.	A	C	T	.	A	T	.	.	.	A	.	.	A	.	.	.	.	.	.	T	T	A	.	.	T	A	T	.	A	G	.	T	.	.	A	.	.	A	.	T	.	A	.	
Allele_43	G	A	.	A	C	T	.	A	T	.	.	.	A	.	.	A	.	.	.	.	.	.	T	T	A	.	.	T	A	T	.	A	G	.	T	.	.	A	.	.	.	.	T	A	A	.	
	S	N	N	S	S	S	S	N	S	S	S	S	N	S	S	N	S	S	N	S	S	S	S	S	N	S	S	S	N	S	S	S	S	S	S	S	S	N	S	S	S	S	S	S	N	S	

Fig. S2. Dendogram presenting the genomic fingerprint pattern of *V. cholerae* O1 and O139 strains isolated from clinical and environmental sources in India. The cluster analysis was carried out using 1.5 % optimization, 1.5 % tolerance and >95 % similarity matrix of Dice similarity coefficient of pulsed-field gel electrophoresis. The dendogram was generated by pulsed-field gel electrophoresis of total chromosomal DNA digested with *NotI* restriction enzyme and correlation between their pulsotype and ST types with respect to their sources and year of isolation.

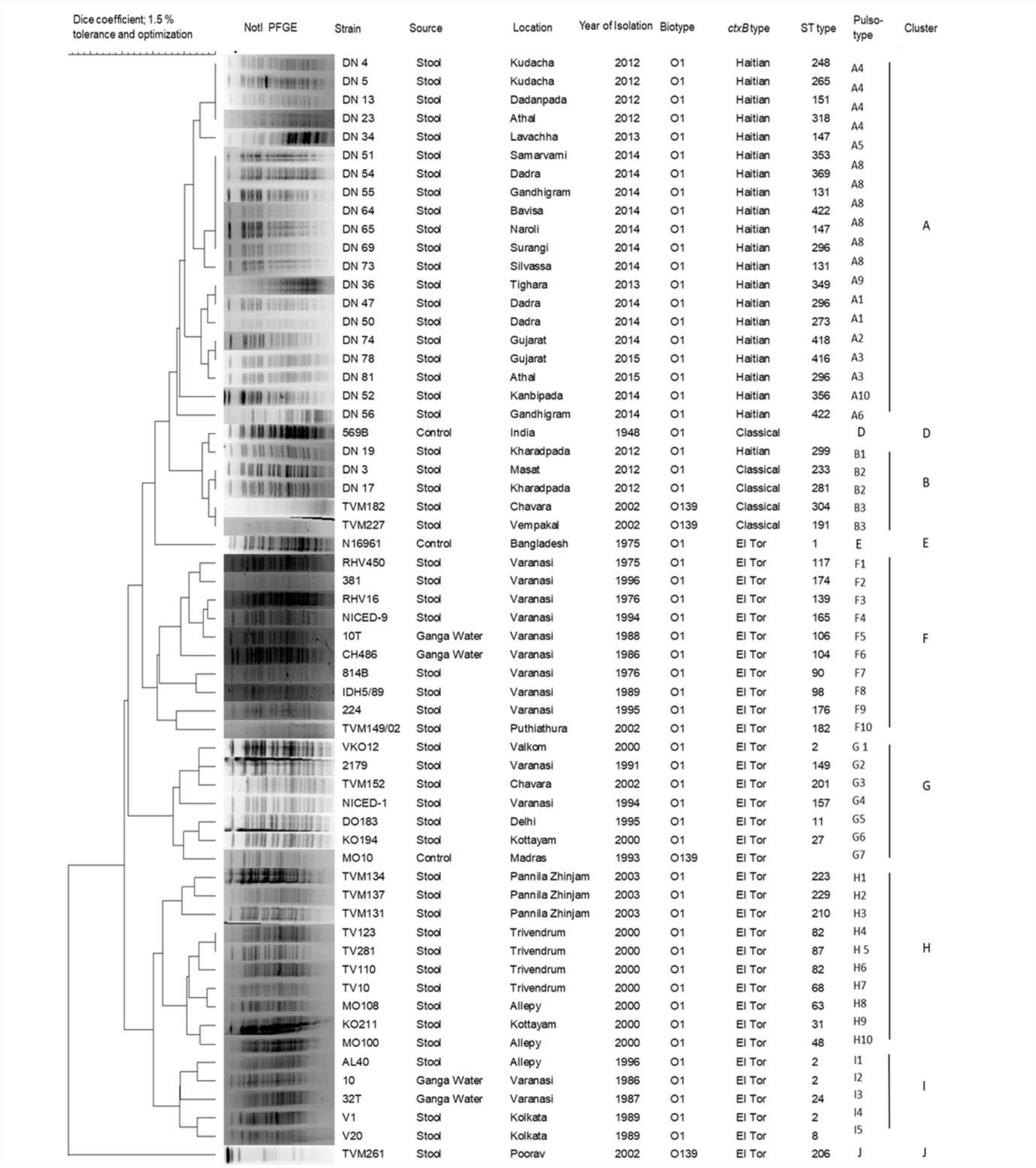

Supplement: Supplementary file 1 [file Data_Sheet_1.pdf]
